# The effects of 12 weeks of chiropractic spinal adjustments on physiological biomarkers in adults: A pragmatic randomized controlled trial

**DOI:** 10.1371/journal.pone.0338730

**Published:** 2025-12-11

**Authors:** Imran Amjad, Imran Khan Niazi, Nitika Kumari, Usman Ghani, Usman Rashid, Felipe Coutinho Kullmann Duarte, Federico Fortuna, Silvia Iglesias, Diego Gonzalez, Alex Sumich, Bibiana Fabre, Kelly Holt, Heidi Haavik

**Affiliations:** 1 Centre for Chiropractic Research, New Zealand College of Chiropractic, Auckland, New Zealand; 2 Faculty of Rehabilitation and Allied Health Sciences, Riphah International University, Islamabad, Pakistan; 3 Faculty of Health & Environmental Sciences, Health & Rehabilitation Research Institute, Auckland University of Technology, Auckland, New Zealand; 4 Centre for Sensory-Motor Interactions, Department of Health Science and Technology, Aalborg University, Aalborg, Denmark; 5 School of Health, Medical and Applied Sciences, CQ University, Rockhampton, Queensland, Australia; 6 Universidad de Buenos Aires, Facultad de Farmacia y Bioquímica, Instituto de Fisiopatología y Bioquímica Clínica (INFIBIOC), Ciudad Autónoma de Buenos Aires, Buenos Aires, Argentina; 7 NTU Psychology School of Social Sciences, City Campus, Nottingham Trent University, Nottingham, United Kingdom; Mayo Clinic College of Medicine and Science, UNITED STATES OF AMERICA

## Abstract

**Background:**

Longer-term effects of chiropractic care on neuroplasticity, stress, and immune biomarkers remain unclear.

**Objective:**

This study evaluates the effects of chiropractic care on physiological biomarkers, including brain-derived neurotrophic factor (BDNF), cortisol (saliva, blood, hair), and inflammatory cytokines [interleukin-6 (IL-6), tumor necrosis factor-alpha (TNF-α), interferon-gamma (IFN-γ), C-reactive protein (CRP), B-lymphocytes (CD19), T-helper cells (CD4), cytotoxic T cells (CD8), and natural killer cells (CD56)] in subclinical spinal pain patients.

**Methods:**

Parallel-group, pragmatic randomized controlled trial conducted at the Rehabilitation Center of Railway General Hospital, Rawalpindi, Pakistan. Intervention: 12 weeks; follow-up: 16 weeks (May–December 2022). Participants with subclinical spinal pain were randomly assigned by using simple lottery method to either 12 weeks of chiropractic or sham care. We aimed to recruit up to 150 participants over three months; however, given the pragmatic nature of the trial and logistical constraints, including the availability of chiropractors, the final number enrolled was determined by how many eligible participants could be recruited during this time. Adults aged 20–60 years with subclinical spinal pain (n = 106 randomized; 88 completed 12-week measures; 73 completed 16-week follow-up). Among those who finished 12 weeks: chiropractic, 26 males/15 females, mean age 37.49 ± 12.39 years; sham, 24 males/23 females, mean age 26.85 ± 7.13 years. The primary outcome blood BDNF and secondary outcome, including saliva, blood and hair cortisol, IL-6, TNF-α, IFN-γ, CRP, CD19, CD4, CD8, and CD56 levels were measured at baseline, after 12 weeks of intervention, and at a 16-week follow-up. Linear and linear mixed-effects regression models were used to assess the effect of care and time on biological measures.

**Results:**

Significant between-group differences were observed after 12 weeks of intervention, with higher salivary cortisol 5 ± 2 [0, 10], p = 0.045 and blood BDNF150 ± 60 (40, 270), p = 0.009 and IL-6 1.0 ± 0.3 [0.5, 1.5], p < 0.001 levels in the chiropractic care group. At the 16-week follow-up, blood cortisol −9 ± 4 [−17, −1], p = 0.024, IFN-γ − 22 ± 7 [−35, −9], and TNF-α −2 ± 1 [−5, 0], p = 0.028 levels increased in the sham group. Within-group comparisons showed a non-significant 10 ± 20 [−20, 50], p = 0.439 reduction in hair cortisol levels in the chiropractic group at 12 weeks, along with increased levels of blood cortisol, BDNF, CD8, CD4, IL-6, and CD19.

**Conclusion:**

12 weeks of Chiropractic care modulates biomarkers linked to neuroplasticity, inflammation, and stress. Increases in brain-derived neurotrophic factor and interleukin-6 suggest enhanced neuroplasticity and inflammatory responses, while decreases in tumor necrosis factor-alpha indicate a regulatory effect on systemic inflammation. These findings support the notion that chiropractic care modulates physiological systemic biomarkers, which may underscore its benefits on clinical outcomes.

**Trial registration:**

ClinicalTrials.gov NCT05369156.

## Introduction

Chiropractors specialise in musculoskeletal health, primarily emphasizing the function and disorders of the spine, including spinal pain. During chiropractic care, chiropractors employ various conservative interventions with emphasis on spinal manipulative therapy (SMT), typically administered over multiple sessions [1,2]. Based on previous research, it has been suggested that vertebral subluxations impact central neural function due to a breakdown in central segmental motor control (CSMC problems) that results in altered afferent feedback from muscle spindles in the paraspinal muscles at subluxated levels of the spine [1,3]. This, in turn, is thought to result in maladaptive neural plastic changes in the central nervous system that result in altered sensorimotor control. The chiropractic adjustments are shown to alter brain activity, including sensorimotor, prefrontal and cerebellar function, and improve the ability of peripheral muscles to produce force [1,4–14]. Whilst maladaptive central activity might thus be improved following chiropractic care (CC) [1,3], longer-term effects, and indeed their precise mechanisms, remain unclear.

Although chiropractic care is widely used and its clinical benefits on pain and function are recognized, little is known about its sustained effects on physiological biomarkers. understanding these effects is important to establish the clinical relevance and mechanistic basis of chiropractic interventions.

For example, one feasible mechanism could be through the immune and stress systems; that is, intervention could impact protective mechanisms (e.g., brain-derived Neurotrophic Factor, BDNF) and inflammation, which would be expected to have enduring effects on brain function.

There is also likely a bidirectional relationship between the function of key brain areas and inflammation, which could be manipulated through CC. For example, the prefrontal cortex (PFC) is centrally involved in multimodal sensory integration. Thus, alteration in PFC activity following chiropractic care is associated with improved joint position sense error [15], cortical processing [16,9], reflex excitability [17], reaction time [16], cortical sensorimotor integration [9,10], motor control [10] and lower limb muscle strength [18]. Through its involvement in regulating hypothalamic function and autonomic activity, the PFC also modulates endocrine and immune systems [19–30]. On the other hand, PFC is vulnerable to the damaging effects of stress and chronically raised inflammation [31]. BDNF protects against HPA dysregulation [32], as well as stress- and inflammation-induced neuronal damage [33]. A recent review [34] suggests chiropractic adjustments modulate immune mediators [35,36], at least within the same day [36–40]; although again, longer-term effects are unclear. These findings indicate that chiropractic care can impact neuroplasticity, stress, and immune function through multiple pathways. however, evidence to date is mostly derived from acute or single-session studies in healthy adults, which limits our understanding of whether these changes are sustained or clinically meaningful in patient populations.

Immune status can be assessed by measuring the concentration of several inflammation mediators, such as tumor necrosis factor-alpha (TNF-α), C-reactive protein (CRP), interleukin (IL)-1 and IL-6, which are essential cytokines produced by monocytes and macrophages. Also, IFN-gamma activates the cells involved in the elimination of bacteria, viruses, fungi, and tumor cells – e.g., monocytes, macrophages, cytotoxic T-lymphocytes (CD3), natural killer cells (CD56) – and promotes the generation of antigen-specific IgG2 by B-lymphocytes (CD19). The ratio of T-helper cells (CD4) and Cytotoxic T cells (CD8) is an indicator of long-term activation of the immune system [41]. Moreover, increased levels of glucocorticoids (e.g., cortisol) can alter cytokine levels (such as increasing IL-6 pro-inflammatory cytokines), subsequently altering inflammation levels throughout the body [42]. Cortisol can be measured from hair, blood, and saliva samples. Blood and salivary cortisol provide a snapshot of acute stress, reflecting current circulating levels. In contrast, hair cortisol is considered a marker of chronic stress, as it provides information on cortisol levels over weeks to months [43]. Recent studies [44–48] have highlighted the role of BDNF, cortisol, and cytokines in chronic pain, stress regulation, and healthy aging. however, very few have examined these biomarkers in the context of chiropractic care, particularly using longer-term follow-up designs. this study was therefore undertaken to address this gap and provide contemporary evidence.

Mechanical loading of various body tissues has been shown to modulate the release of pro- and anti-inflammatory cytokines, which are crucial for inflammation and tissue repair processes [49–53]. High-load cyclic compressive massage, in a pre-clinical study, increased macrophage infiltration and elevated muscle inflammation in the healthy tibialis anterior muscle [54]. Macrophages also play a significant role in the release of inflammatory cytokines, contributing to inflammation, intercellular signalling, and tissue repair [55]. Lower loading magnitudes of compressive massage stimulate the M2 subpopulation of macrophages, which are involved in tissue repair and regeneration, as opposed to the M1 subpopulation, which is associated with the secretion of pro-inflammatory cytokines [56]. In another recent study, higher compared to lower SMT force raised plasma pro-inflammatory and dual-role cytokines in healthy, young adults [57]. Taken together, these data suggest that pro- and anti-inflammatory markers are sensitive to manual therapy and that CC might well influence these markers. However, most prior studies have been limited to single sessions or acute outcomes, few have investigated immune cell subset changes (CD4, CD8, CD19, CD56), and almost none have linked biomarker changes to clinical outcomes in patients with spinal pain. these gaps underscore the need for longitudinal, well-controlled studies.

Demonstrating longer-term effects of chiropractic care (CC) on immune and neuroplasticity markers would represent an important advance in understanding the physiological mechanisms underlying its clinical ef-fects. We hypothesized that 12 weeks of chiropractic care would modulate biomarkers related to stress (cor-tisol), neuroplasticity (BDNF), and inflammation (IL-6, TNF-α, IFN-γ, CRP, and immune cell profiles) compared to sham care in adults with subclinical spinal pain. To test this hypothesis, we conducted a prag-matic randomized controlled trial (RCT) examining the effects of CC on a range of blood, saliva, and hair-derived physiological biomarkers over a 16-week period. This study is clinically significant because it offers new mechanistic insights for researchers, provides clinicians with biomarker-informed evidence to guide practice, and demonstrates to patients that chiropractic care may improve neurophysiological resilience, immune balance, and stress regulation beyond symptom relief.

## Materials and methods

### Design and setting

A parallel-group, pragmatic RCT was conducted at the Rehabilitation Center of Railway General Hospital, Rawalpindi, Pakistan (from May to December 2022). The Ethical Review Committee of Riphah International University, Pakistan, approved the study (Riphah/RCRS/REC/01288), which was registered with the National Institutes of Health ClinicalTrials.gov clinical trial registry (NCT05369156; first registration on 05 May 2022). The data collection period was from 15 May to 1 November 2022. All participants provided written informed consent before enrollment in the study.

### Study participants

Participants were included if they were aged between 20 and 60 years and had subclinical spinal pain. Subclinical spinal pain refers to recurrent spinal aches, pain, or stiffness, for which the person has not yet sought treatment [58]. The participants were pain-free at the time of their baseline assessment, i.e., less than or equal to 3/10 on a pain-based visual analogue scale (VAS). People with subclinical pain were recruited because subclinical pain participants are more likely to have altered neurophysiological and biomechanical function than completely pain-free participants [58]. Participants were excluded from the study if any of the following conditions applied: no evidence of spinal dysfunction is present, they are in current pain (above 3/10 on VAS), they had sought previous treatment for their spinal issues, they were unable to perform the assessment procedures due to contra-indications or movement limitations, were diagnosed with immune dysfunction and/or utilized a prescribed immunosuppressive medication. Participants were also excluded if they had uncontrolled asthma, had nasal polyps, used of an intranasal steroid spray one month or less before the study, were HIV-positive, had abnormal laboratory values (i.e., any change from reference values in the last 12 months) for complete blood count or hormonal changes, were pregnant or breastfeeding women, had a history of drug abuse, was unable or unwilling to comply with the study protocol (i.e., relating to blood draws), were participating in another research study during the time of data collection, had any diagnosed comorbidity or concomitant disease (diabetes, hypertension or any neurological condition), had donated blood within last month, had allergies to yeast or yeast-derived products, or had chronic sinusitis and/ or recent (within the last six weeks) episode of acute sinusitis. Written consent was obtained from all volunteers before they participated in the study.

### Sample size

The sample size was calculated based on 80% power and a 95% significance level. A total of 150 participants was sufficient to detect an effect size of 0.25 in the tests of multimodal data included as primary outcome measures in this study [59]. We planned to allocate 75 participants to the chiropractic care (CC) group and 75 to the control group. Recruitment was open for a maximum of three months, during which we aimed to enrol as many eligible participants as possible, to reach 150 participants. Recruitment was limited to a fixed three-month period due to the availability of chiropractors to deliver the intervention.

### Interventions

The interventions were 12 weeks of chiropractic and 12 weeks of sham chiropractic care. Participants were allowed to receive usual care if required as part of the best ethical practice to ensure symptom relief, avoid withholding standard care, and improve the acceptability of participation.

#### Chiropractic intervention.

In the chiropractic group, a New Zealand registered chiropractor checked participants for CSMC problems and delivered chiropractic adjustments to the appropriate segments during the experimental sessions.. Participants were checked by the chiropractor approximately three times per week for 12 weeks, with interventions delivered pragmatically to reflect routine clinical practice, allowing adjustments to be tailored based on individual clinical assessments. At each experimental session the chiropractor assessed the participant’s spine for indicators of CSMC problems. These indicators are routinely used by chiropractors to determine the appropriate segments of the spine and pelvis that require chiropractic adjustments and, when used in combination, have been shown to be reliable indicators for determining CSMC problems [60,61]. The indicators used include static and motion palpation to assess for spinal tenderness, restricted intersegmental motion, increased muscle tension and reduced joint play. Once the site of the CSMC problem has been identified the chiropractor delivers the adjustments either with a manual, high-velocity, low amplitude thrust (HVLA) or with an instrument-assisted thrust [62]. Typically, multiple sites of the spine were adjusted at each experimental session, as deemed appropriate by the chiropractor based on the spinal assessment. Each chiropractic visit lasted approximately 15–20 minutes.

#### Sham (Control) chiropractic intervention.

Research involving physical or manual interventions can face challenges with the blinding of participants [63,64]. Whilst it is difficult to fully blind participants in a study of this nature, it is possible to control contextual factors to enhance the chances of successful blinding. This study was conducted in Pakistan where chiropractic care is relatively unknown [36]. In a recent survey of university students in Lahore, Pakistan, including pharmacy students, more than two-thirds of respondents were unaware that chiropractic care involved spinal manipulation and that it is used as an intervention for low back pain [65]. The limited understanding of chiropractic care presents a unique opportunity to investigate its effects while maximizing the chances of successful participant blinding. By carefully controlling contextual factors in this study design, we can more effectively isolate and analyze the specific contributions of chiropractic treatment beyond those of placebo and contextual effects. The control group, in this case, received a sham chiropractic intervention.

This involves participants attending experimental sessions at the same frequency and duration as the intervention group. At these sessions the same chiropractor performed a spinal assessment but no chiropractic adjustments are performed. Instead, the chiropractor would position the patient as they normally would to deliver the adjustment, but with no thrust on the spine. Alternatively, the chiropractor would use the adjusting instrument on a low setting to produce the clicking sound, but with no contact on the participants spine.

### Outcome measures

All outcome measures were assessed at baseline (before the first chiropractic session), 12 weeks (post-intervention), and at 16 weeks (to assess retention effects). Although multiple measures were collected as part of the broader study, this paper specifically focuses on blood-based, saliva and Hair markers. The primary outcome measure was the blood BDNF levels. Secondary outcome measures included the B-Lymphocytes (CD19), CD4, CD8, IL-6, TNF-alpha, C-reactive protein (CRP), interferon-gamma, Natural killer cells (CD56) and blood, hair, and saliva cortisol. Following the 16-week (follow-up session), participants in both groups were asked to indicate whether they thought they had received active chiropractic care using a yes or no response to test the effectiveness of participant blinding.

Blood samples were collected from participants via venipuncture into sterile blood separation anticoagulant tubes. A total of 5 mL of blood was drawn from each participant. After collection, the samples were allowed to clot at room temperature for 30 minutes and were subsequently centrifuged at 3000 x g for 10 minutes to separate the blood. The blood was divided into two microcentrifuge tubes and immediately stored at −80°C until analysis. Each participant was provided with a labelled plastic container for saliva collection and instructed to collect their saliva early in the morning, before 7 o’clock. These containers were then stored at −80°C. Each sample underwent one freeze-thaw cycle before analysis to minimize the degradation of biomarkers. Hair samples were collected by selecting a small section of hair from the vertex area, cutting it with scissors, and placing it in aluminium foil. The samples were then stored at room temperature.

Blood concentrations of biomarkers (explained earlier) and cortisol concentration from saliva were evaluated using commercially available enzyme-linked immunosorbent assay (ELISA) kits (abcam, Elabscience, ELK Biotechnology) following the manufacturer’s instructions. The optical density (OD) was measured using a microplate reader (RaytoRC 2100 C, China) at a wavelength of 450 nm. The samples were analyzed at Health Plus Lab, Rawalpindi, Pakistan. Calibration curves were generated for each assay, and sample concentrations were calculated using a four-parameter logistic (4PL) curve-fitting model provided by the ELISA kit software.

Assay reliability was confirmed by intra- and inter-assay coefficients of variation consistently <10–15% across all kits. Each plate included standardised controls and calibration curves for quantification. Validity was further supported by manufacturer data, which confirmed high specificity with no significant cross-reactivity (<5%) and reproducibility across various biological matrices.

Analytical sensitivity (LOD/LLOQ) varied by biomarker, ranging from 0.19 ng/mL for CD4, 0.23 ng/mL for CRP, and 0.118 ng/mL for CD19 to 18.75 pg/mL for BDNF. Detection ranges spanned several orders of magnitude, for example: Cortisol 6.25–400 ng/Ml; IFN-γ 15.63–1000 pg/mL; TNF-α 7.81–500 pg/mL; CD8 1.25–80 ng/mL; IL-6 1.56–100 pg/mL CD56 1.56–100 ng/mL; CD19 0.32–20 ng/mL Recovery rates for serum and plasma samples were typically within ~90–110%, in line with immunoassay validation guidelines, supporting the accuracy of quantification.

Hair samples were collected using scissors from the posterior vertex, as close to the scalp as possible, to evaluate the hair cortisol concentration. Each sample was stored in a paper envelope at room temperature. Cortisol was extracted and quantified using an automated chemiluminescent method (Immulite 2000 autoanalyzer, Siemens, LA, USA) following the protocol reported by Gonzalez et al. Hair cortisol concentration was expressed in pg/mg of hair [66].

Potential harms or adverse events were investigated by asking participants, during scheduled intervention visits, about any injuries or perceived adverse effects of care that may have occurred during the trial, such as muscle soreness or pain [67].

### Randomization and blinding

Randomization was carried out following the baseline assessment by a simple lottery method. All participants and the outcome assessors were blinded to group allocation. An investigator undertook and collated all outcome measurements and remained blinded to participant allocation throughout the study, the outcome assessor was not involved in administering the interventions. The statistician who analyzed the data was also blinded to group allocation, as all recorded data were anonymized and coded before being provided for analysis. The chiropractors providing chiropractic care could not be blinded to group allocation.

### Statistical analysis

The primary null hypothesis for the analysis was that there was no difference in blood BDNF levels between the chiropractic and sham groups. Pre-specified secondary null hypotheses stated that there were no differences between the two groups in CD19, CD4, CD8, IL-6, TNF-alpha, CRP, interferon-gamma, CD56 and blood and saliva cortisol.

Linear and linear mixed effects regression models were constructed to analyze the pre- to post-intervention change scores of the outcome measures. Multiple outcome measures were included in a single model if they were correlated and had similar error variance. This multivariate analysis of covariance using linear mixed effects modelling is statistically superior to separate models for each outcome as it results in better parameter estimation [68].The models regressed the change scores on the pre-intervention values, group, and time in the case of outcomes with follow-up, outcome, and interactions between these fixed factors. The within-participant correlations arising from repeated measures were accounted for by including participant-level random intercepts in the models. Where a model included multiple outcomes, these random intercepts were estimated for each outcome. The linear regression model was only fitted to the hair cortisol as it was only measured at pre- and post-intervention time points. Linear mixed effects regression was used for all the other outcomes. The assumptions of normality and homogeneity of error variance were evaluated with QQ-plots and errors-versus-fitted values plots. A uniform dispersion of the residuals around the zero line for all fitted values was the expected norm. Any deviations from this norm, such as a fan-out or fan-in pattern, were taken as an indication of poor model fit to the data. In such cases, a separate model was fitted for each outcome, rather than pooling multiple outcomes in a single model. The model parameters are used to estimate between and within-group differences at successive time points. These mean differences in change scores were presented with their standard errors, 95% confidence intervals and hypothesis test statistics (t-values, degrees of freedom, p-values) for 0 change at 5% type-I error rate.

To test these hypotheses, data collected during the study were collated in an Excel (Microsoft Corp., Redmond, WA) spreadsheet with groups relabeled for blinding. Statistical analyses were conducted in R version 4.0.0 using the packages lme4 [69–71]. A detailed report of the statistical analysis is available in the supplementary file(S1 File).

Mean differences for between-group and within-group differences are reported with standard errors and 95% confidence intervals that were obtained from the models. P-values for testing the primary and secondary null hypotheses were obtained with z or t-tests based on these estimates. Cohen’s *d* corresponding to the mean differences is also reported. The statistical significance level was set at 0.05. Šidák’s correction was applied to multiple comparisons from the same model to control the family-wise error rate for correlated outcomes and timepoints.

Due to the difference in the number of participants willing to give samples for different tests after group allocation, a per-protocol analysis was performed. Nonetheless, to evaluate the sensitivity of the statistically significant results to missing data, the primary models were refit with multiple imputation for missing values [72]. Statistically significant results that remained statistically significant in the sensitivity analysis were marked with an obelisk (†).

## Results

**Fig 1:**
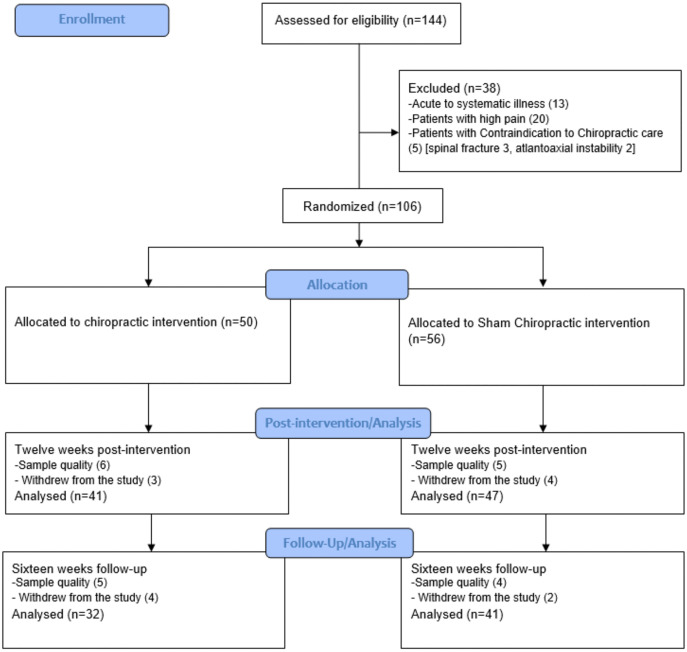
CONSORT study flow diagram.

One hundred forty-four volunteers were recruited and checked for eligibility during a three-month recruitment period. 106 participants met the inclusion criteria and agreed to participate. 88 completed the post-12-week measure(n = 41 in chiro group,n = 47 in sham group), and 73 completed the 16-week (n = 32 in chiro group,n = 41 in sham group) follow-up session (Fig 1, CONSORT diagram). A few participants (n = 5) reported mild transient muscle soreness and stiffness in the back muscles following the first intervention visit in the interventional group, resolving spontaneously within 24 hours without intervention; otherwise, no adverse events or reports of harm were received during the trial. Participants’ clinical characteristics are given in Table 1, who finished 12 weeks post-intervention.

**Table 1:** Clinical characteristics of participants in each group.

Variables	Chiropractic	Sham Chiropractic
**Gender**		
Male (n)	26	24
Female (n)	15	23
**Age, years (mean + /- SD)**	37.49 + /-12.39	26.85 + /- 7.13

n: number of participants, SD: standard deviation.

### Participant blinding

After the 16-week intervention period, all participants who completed the trial were asked if they thought they had received active chiropractic care. 39 of the 41 participants in the chiro group and 43 of the 47 participants in the sham group believed they had received active chiropractic care. This suggests that participant blinding was successful, with 94% of participants across the two groups believing they had received active chiropractic care, with no between-group differences present.

**Fig 2:**
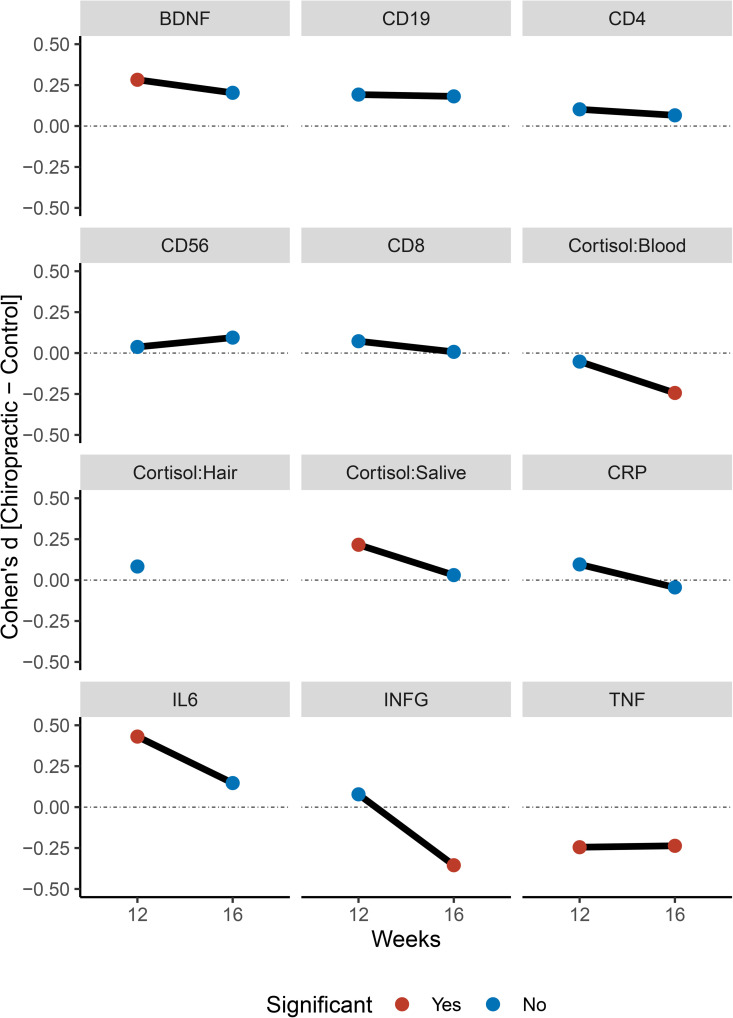
The first point shows the difference between the groups after the intervention, and the second point shows the difference after the follow-up period. Positive values indicate that the chiropractic group has higher values, while negative values indicate that the control group has higher values.

Fig 2 shows line graphs representing group differences at 12 and 16 weeks. Table 2 shows t-statistics for between-group comparisons and 12 and 16 weeks. Table 3 shows t-statistics for within-subjects effects.

**Table 2:** Between-group comparison. Timepoint 12 = After 12 weeks of intervention, Timepoint 16 = After 16 weeks for follow-up, * Significant in per-protocol analysis, ^†^ remained statistically significant in the sensitivity analysis.

Variable	Time Point	Contrast	Difference±SE [95% CI], SMD	t[df], p-value
BDNF	12	Chiropractic – Control	150 ± 60 [40, 270], 0.21	t[156.8]=2.65, 0.009*†
	16	Chiropractic – Control	120 ± 60 [0, 250], 0.15	t[156.9]=1.904, 0.059
Hair Cortisol	12	Chiropractic – Control	10 ± 20 [−20, 50], 0.1	t[62]=0.779, 0.439
Blood Cortisol	12	Chiropractic – Control	−2 ± 4 [−9, 6], −0.05	t[109.5]=−0.488, 0.627
	16	Chiropractic – Control	−9 ± 4 [−17, −1], −0.2	t[125.9]=−2.286, 0.024*
Saliva Cortisol	12	Chiropractic – Control	5 ± 2 [0, 10], 0.16	t[154.1]=2.024, 0.045*†
	16	Chiropractic – Control	1 ± 3 [−5, 6], 0.02	t[176.8]=0.286, 0.775
INF-γ	12	Chiropractic – Control	4 ± 6 [−8, 17], 0.07	t[123.6]=0.732, 0.465
	16	Chiropractic – Control	−22 ± 7 [−35, −9], −0.28	t[137]=−3.331, 0.001*†
CRP	12	Chiropractic – Control	1 ± 1 [−1, 3], 0.08	t[122.1]=0.898, 0.371
	16	Chiropractic – Control	0 ± 1 [−3, 2], −0.04	t[140.9]=−0.421, 0.675
TNF-α	12	Chiropractic – Control	−2 ± 1 [−4, 0], −0.21	t[124.2]=−2.298, 0.023*†
	16	Chiropractic – Control	−2 ± 1 [−5, 0], −0.18	t[145.6]=−2.218, 0.028*
IL6	12	Chiropractic – Control	1 ± 0.3 [0.5, 1.5], 0.34	t[144.2]=4.041, < 0.001*†
	16	Chiropractic – Control	0.4 ± 0.3 [−0.2, 0.9], 0.11	t[163]=1.377, 0.17
CD19	12	Chiropractic – Control	0.5 ± 0.3 [0, 1], 0.15	t[143.6]=1.803, 0.074
	16	Chiropractic – Control	0.5 ± 0.3 [−0.1, 1], 0.13	t[163.3]=1.701, 0.091
CD4	12	Chiropractic – Control	1 ± 1 [−1, 3], 0.09	t[117.6]=0.958, 0.34
	16	Chiropractic – Control	1 ± 1 [−2, 3], 0.05	t[135.6]=0.614, 0.54
CD8	12	Chiropractic – Control	2 ± 3 [−4, 9], 0.06	t[141.3]=0.679, 0.498
	16	Chiropractic – Control	0 ± 4 [−7, 8], 0.01	t[148.6]=0.069, 0.945
CD56	12	Chiropractic – Control	0 ± 1 [−2, 3], 0.03	t[125.4]=0.35, 0.727
	16	Chiropractic – Control	1 ± 1 [−1, 4], 0.08	t[139.1]=0.885, 0.378

**Table 3:** Within-group comparison. Timepoint 12 = After 12 weeks of intervention, Timepoint 16 = After 16 weeks for follow-up, * Significant in per-protocol analysis, ^†^ remained statistically significant in the sensitivity analysis.

Variable	Time Point	Group	Estimate±SE [95% CI], SMD	t[df], p-value
BDNF	12	Chiropractic	80 ± 40 [−10, 180], 0.16	t[156.8]=1.976, 0.097
	12	Control	−70 ± 40 [−160, 20], −0.14	t[156.8]=−1.767, 0.152
	16	Chiropractic	70 ± 50 [−30, 180], 0.13	t[156.9]=1.572, 0.222
	16	Control	−50 ± 40 [−140, 50], −0.09	t[156.9]=−1.105, 0.469
Hair Cortisol	12	Chiropractic	20 ± 10 [−10, 50], 0.23	t[62]=1.841, 0.136
	12	Control	10 ± 10 [−20, 40], 0.09	t[62]=0.674, 0.753
Blood Cortisol	12	Chiropractic	16 ± 3 [9, 23], 0.51	t[115.3]=5.453, < 0.001*^†^
	12	Control	18 ± 3 [11, 24], 0.58	t[117.2]=6.271, < 0.001*^†^
	16	Chiropractic	6 ± 3 [−1, 13], 0.18	t[135.4]=2.039, 0.085
	16	Control	15 ± 3 [9, 22], 0.47	t[124.9]=5.28, < 0.001*^†^
Saliva Cortisol	12	Chiropractic	6 ± 2 [1, 10], 0.18	t[213.9]=2.698, 0.015*^†^
	12	Control	1 ± 2 [−4, 5], 0.03	t[215.6]=0.404, 0.902
	16	Chiropractic	0 ± 2 [−5, 6], 0.01	t[249.2]=0.172, 0.981
	16	Control	0 ± 2 [−5, 5], −0.01	t[237.5]=−0.172, 0.981
INF-γ	12	Chiropractic	10 ± 4 [0, 20], 0.21	t[123.7]=2.318, 0.044*^†^
	12	Control	6 ± 4 [−4, 15], 0.13	t[124]=1.393, 0.305
	16	Chiropractic	0 ± 5 [−11, 11], 0.01	t[140.4]=0.076, 0.996
	16	Control	22 ± 4 [12, 32], 0.44	t[132.8]=5.089, < 0.001*^†^
CRP	12	Chiropractic	1.6 ± 0.8 [−0.2, 3.4], 0.18	t[134.3]=2.066, 0.08*
	12	Control	0.7 ± 0.7 [−1, 2.4], 0.08	t[131.3]=0.941, 0.575
	16	Chiropractic	−0.6 ± 0.8 [−2.5, 1.3], −0.06	t[157.9]=−0.756, 0.699
	16	Control	−0.2 ± 0.8 [−1.9, 1.5], −0.02	t[141.3]=−0.229, 0.967
TNF-α	12	Chiropractic	1 ± 0.8 [−0.8, 2.8], 0.11	t[131.9]=1.267, 0.372
	12	Control	3.3 ± 0.8 [1.6, 5], 0.38	t[132.3]=4.376, < 0.001*^†^
	16	Chiropractic	1.7 ± 0.9 [−0.3, 3.6], 0.15	t[159.3]=1.918, 0.111
	16	Control	4 ± 0.8 [2.3, 5.8], 0.42	t[146.8]=5.116, < 0.001*^†^
IL6	12	Chiropractic	1 ± 0.2 [0.6, 1.4], 0.45	t[145.4]=5.391, < 0.001*^†^
	12	Control	0 ± 0.2 [−0.4, 0.4], −0.01	t[145]=−0.101, 0.994
	16	Chiropractic	0.7 ± 0.2 [0.2, 1.1], 0.25	t[169.9]=3.266, 0.003*
	16	Control	0.3 ± 0.2 [−0.1, 0.7], 0.13	t[155.6]=1.628, 0.2
CD19	12	Chiropractic	0.4 ± 0.2 [0, 0.8], 0.19	t[144.1]=2.282, 0.047*
	12	Control	0 ± 0.2 [−0.4, 0.4], −0.02	t[148.9]=−0.203, 0.974
	16	Chiropractic	0.1 ± 0.2 [−0.3, 0.6], 0.05	t[168.2]=0.688, 0.742
	16	Control	−0.3 ± 0.2 [−0.7, 0.1], −0.14	t[162.4]=−1.755, 0.156
CD4	12	Chiropractic	2.2 ± 0.8 [0.4, 4], 0.24	t[123.8]=2.696, 0.016*
	12	Control	1.1 ± 0.8 [−0.6, 2.9], 0.14	t[122.2]=1.496, 0.256
	16	Chiropractic	1.2 ± 0.9 [−0.8, 3.1], 0.11	t[147.2]=1.353, 0.325
	16	Control	0.5 ± 0.8 [−1.3, 2.2], 0.05	t[132.1]=0.592, 0.802
CD8	12	Chiropractic	4 ± 2 [−1, 10], 0.15	t[141.3]=1.829, 0.134
	12	Control	2 ± 2 [−3, 7], 0.08	t[141.4]=0.949, 0.57
	16	Chiropractic	4 ± 3 [−2, 10], 0.12	t[150.3]=1.459, 0.272
	16	Control	4 ± 2 [−2, 9], 0.13	t[146.2]=1.541, 0.235
CD56	12	Chiropractic	1.2 ± 0.9 [−0.8, 3.1], 0.12	t[125.7]=1.35, 0.327
	12	Control	0.7 ± 0.8 [−1.1, 2.6], 0.08	t[125.4]=0.914, 0.593
	16	Chiropractic	1.7 ± 1 [−0.4, 3.9], 0.15	t[143]=1.793, 0.145
	16	Control	0.6 ± 0.9 [−1.4, 2.5], 0.06	t[133.5]=0.671, 0.753

### Blood BDNF concentration

There was a significant effect of *Group* for BDNF levels (Chiro>Sham) at 12 weeks (mean difference = 150 ± 60; 95% CI: 40–270; *p* = 0.009), but this effect failed to reach significance at 16 weeks (120 ± 60; 95% CI: 0–250; *p* = 0.059). Within-group comparison shows a significant increase in blood BDNF levels in the chiropractic care group after 12 weeks (80 ± 40; 95% CI: 0–170; *p* = 0.050), compared to the control group. At the 16-week follow-up, there is no significant difference between the chiropractic care group and the control group.

### Blood, hair and saliva cortisol

No significant between-group difference for serum cortisol at 12 weeks (−2 ± 4; 95% CI: −9–6; *p* = 0.627). At 16 weeks, the chiropractic group showed significantly lower serum cortisol (−9 ± 4; 95% CI: −17 to −1; *p* = 0.024).

For hair cortisol no significant difference at 12 weeks (10 ± 20; 95% CI: −20–50; *p* = 0.439). A within-group trend toward reduction was observed in the chiropractic group at 12 weeks (20 ± 10; 95% CI: 0–50; *p* = 0.070). At 12 weeks, salivary cortisol was higher in the chiropractic group (5 ± 2; 95% CI: 0–10; *p* = 0.045), but no significant effect was found at 16 weeks (1 ± 3; 95% CI: −5–6; *p* = 0.775).

### Blood CD19, CD4, CD56, and CD8

There were no significant effects of *Group* for CD19, CD4, CD56, or CD8 at 12 weeks or 16 weeks. However, there was a significant increase in CD19, CD4, and CD8 in the chiropractic care group at 12 weeks, which was not seen in the sham group. But CD56 showed a significant increase in the chiropractic care group. There was no significant effect of *Group* or *Time* for CD19, CD4, CD56, or CD8 at 16 weeks.

### Blood CRP, IL-6, INF-γ and TNF-α concentration

No significant effects of *Group* in CRP were seen at either 12 (1 ± 1; 95% CI: −1–3; *p* = 0.371) or 16 weeks (0 ± 1; 95% CI: −3–2; *p* = 0.675). However, there was a significant increase between baseline and 12 weeks, with no further change between 12 and 16 weeks. The chiropractic care group showed significantly higher IL-6 than Sham at 12 weeks(1.0 ± 0.3; 95% CI: 0.5 to 1.5; *p* < 0.001), but not at 16 weeks (0.4 ± 0.3; 95% CI: −0.2 to 0.9; *p* = 0.170). No effect on blood INF-γ levels at 12 weeks (4 ± 6; 95% CI: −8–17; *p* = 0.465), but significantly lower in the chiropractic group at 16 weeks (−22 ± 7; 95% CI: −35 to −9; *p* = 0.001). This appeared to be due to a significant increase in the Sham group between 12 and 16 weeks. There was a significant lower TNF-alpha at 12 (−2 ± 1; 95% CI: −4–0; *p* = 0.023) and 16 weeks (−2 ± 1; 95% CI: −5–0; *p* = 0.028) in the chiropractic group.

## Discussion

This is the first investigation of the longer-term effects of Chiropractic care on biological markers of neuroplasticity, stress-response and immune system function. In line with hypotheses, present findings demonstrate Chiropractic care affects BDNF levels (higher in the chiropractic care group), blood TNF-α levels (lower in the chiropractic care group) and saliva cortisol levels (higher in the chiropractic care group) after 12 weeks. Additionally, at the 16-week follow-up, there were significant differences in blood cortisol levels (higher in the sham group), blood INF-γ levels (higher in the Sham group), and blood TNF-α levels (higher in the Sham group). Within-group comparisons showed a significant reduction in hair cortisol levels in the chiropractic care group after 12 weeks, while blood cortisol, saliva cortisol, blood BDNF, CD8, CD4, INF-γ, TNF-α, IL-6, and CD19 levels increased significantly in the chiropractic care group after 12 weeks. There were no significant differences between groups or within groups for blood CD8, CD4, CD56, or CRP levels at both time points. The relatively small effect sizes and wide confidence intervals for some biomarkers (e.g., cortisol, IFN-γ) suggest that while trends were observed, the intervention’s effectiveness may be modest, and results should be interpreted with caution. This variability highlights the need for larger trials with sufficient power. Although we anticipated that changes in immune and stress biomarkers may be partly mediated by altered central neural function, direct measures of brain activity were not reported in this study. Our previous studies have shown changes in cortical excitability and prefrontal activation following CC [9,13], providing initial evidence that neuroimmune modulation may cause such effects. In future, we plan to integrate neurophysiological and biomarker measures to formally test this mediation hypothesis using structural equation or multilevel mediation models.

The increase in BDNF levels observed in the chiropractic group suggests enhanced neuroplasticity compared to the control group, indicating that chiropractic intervention may stimulate neuroplastic activity in the central nervous system (CNS) [73]. BDNF, part of the neurotrophin family along with nerve growth factor (NGF), is crucial for the initiation and maintenance of CNS adaptations [74]. For instance, previous evidence studying the effects of spinal manipulation on growth factors observed that spinal manipulation, commonly used in chiropractic practice, stretches spinal muscles and stimulates the mechano-growth factor (MGF), a splice product of the insulin-like growth factor (IGF-1) gene. MGF is involved in muscle and neuronal growth and repair [75]. Furthermore, non-noxious mechanical skin stimulation has been reported to stimulate NGF release from cortical neurons, which plays a key role in neuronal survival and function [76]. These findings underscore the potential for chiropractic interventions to influence neurotrophic factors and promote neuroplasticity, thereby enhancing neuronal health and function [77].

The chiropractic intervention also resulted in higher serum levels of IL-6 after 12 weeks. Other inflammatory cytokines, such as TNF-α, were significantly higher in the sham group compared to the chiropractic group at both 12 and 16 weeks, and IFN-γ was higher in the sham group at 16 weeks. Although IL-6 is well known to be released in large amounts during infection, autoimmunity and cancer, it is also released by skeletal muscles during exercise [78]. When muscles are used intensely, they release IL-6, in which case it is considered a myokine, where it plays a role in local regulation of the inflammatory process and skeletal muscle regeneration [78]. Thus, the changes in inflammatory markers found in the current study may be explained in the following manner. It has been documented that deep paraspinal muscles undergo numerous maladaptive plastic processes over time following an injury, including muscle atrophy, fatty infiltration, changes in fiber type and morphological changes of the muscle spindles [79–83]. Thus, the chiropractic intervention in this study, which consisted of repeated HVLA adjustments of such dysfunctional spinal segments, may be considered akin to a kind of exercise for these deep paraspinal muscles. The HVLA intervention used as part of the chiropractic intervention rapidly stretches these deep muscles and is known to produce ongoing increased intervertebral range of motion at that segment for a time after the adjustment [84]. Thus, the increase in IL-6 found in this study may reflect an increased release of this myokine in response to the repeated HVLA adjustment provided over the 12 weeks. As the adjustments stopped at 12 weeks, the release of IL-6 would naturally be reduced by 16 weeks; hence, we found no significant difference in IL-6 levels at 16 weeks. IFN-γ and TNF-α are produced by immune cells (like macrophages and T cells) and non-immune cells (such as fibroblasts and endothelial cells) [57]. They are known to play pro-inflammatory and signalling roles, while others, like IL-4 and IL-10, have anti-inflammatory effects. Interestingly, these pro-inflammatory cytokines were not increased in the chiropractic group at either the 12-week mark or the 16-week mark. However, they were significantly higher in the sham group, suggesting the sham group, which did not receive CC, was steadily getting worse (more inflamed) over the study period. This finding supports the notion that the IL-6 changes seen in the chiropractic group at 12 weeks most likely reflect skeletal muscle regeneration.

The cortisol changes observed in this study were interesting. In the current study, the saliva cortisol levels were significantly increased in the chiropractic group at 12 weeks, yet the serum cortisol levels were significantly decreased at 16 weeks. Cortisol generally rises following acute injury to modulate and limit the initial pro-inflammatory response [85]. In the context of CC, the increase in saliva cortisol at 12 weeks could be a response to the mechanical stress and strain associated with the HVLA chiropractic adjustments [57,75–77]. Interestingly, the overall serum cortisol levels at 16 weeks ended up lower than at baseline, suggesting that overall inflammation levels were significantly reduced 4 weeks after the 12-week intervention had stopped. This finding is in line with the IFN-γ and TNF-α immune markers as well. IFN-γ plays a role in activating the immune system in response to damaged cells. As there was no increase in IFN-γ in the chiropractic group, while this significantly increased in the sham group, this suggests that chiropractic care does not damage cells and is more likely to be linked to exercise for the deep paraspinal muscles surrounding the vertebral subluxations. The elevated cortisol levels observed after the chiropractic intervention may be due to an influence of the neuroimmunomodulation pathway and the influence of the HPA axis [86,87]. Increased cortisol levels could suppress systemic inflammatory agents like TNF-α, helping fine-tune immune and inflammatory activity during tissue repair [86]. This complex interplay highlights the body’s regulatory mechanisms in balancing inflammation and immune response following chiropractic care.

After 12 weeks of intervention, there were no significant changes in CRP. When IL-6 is released as a pro-inflammatory marker, it regulates the acute release of CRP from the liver. Yet the current study did not find an increase in CRP following the chiropractic intervention. Thus, following 12 weeks of chiropractic care, it is more likely that the increase seen in the IL-6 may be due to its release as myokines in the deep paraspinal muscles following the repeated HVLA adjustments. This notion is further supported by the finding of TNF-α levels being significantly less in the chiropractic group at both the post-12-week intervention period and at the post-16-week time point. This reduction in TNF-α levels is also consistent with previous studies showing that chiropractic care, including SMT, can lead to decreased TNF-α levels [88–91]. TNF-α plays a crucial role in initiating and propagating acute inflammation. Its reduction could result from a negative feedback mechanism in response to the initial acute inflammatory reaction, aiming to modulate overall systemic inflammation, which is largely mediated by TNF-α [92].

### Strengths and limitations

Statistically significant between-group differences were observed in BDNF, IL-6, TNF, and salivary cortisol levels at the 12-week mark with a sample size of 88 participants. These results suggest that the study was adequately powered for these specific outcome measures at the primary endpoint. The study was designed pragmatically to recruit up to 150 participants; however, the final enrollment (n = 106 randomized) reflected real-world feasibility constraints, including the availability of chiropractors within a fixed three-month recruitment window. However, we acknowledge that this sample size may have been insufficient to detect between-group differences in other outcome measures. Consequently, the possibility of type II errors cannot be ruled out. As an exploratory study, we employed a wide range of outcome measures and performed multiple comparisons without applying adjustments to p-values. While this approach increases the likelihood of type I errors, it is considered appropriate when investigating novel areas of research, such as the present study [93]. Nevertheless, this remains a limitation that should be addressed in future studies. Moreover, the relationship and implications of the expression of these physiological markers should be evaluated in relation to clinical outcomes. In the current study, we also have some data on clinical outcomes, and we plan to conduct a secondary analysis on this.

One strength of the current study is the use of a broad array of inflammatory markers, which provided a more comprehensive understanding of the effects of chiropractic care on physiological markers. This approach will allow for a more detailed and specific evaluation of the mechanisms behind the effectiveness of chiropractic interventions. Future large-scale RCTs should target more specific mediators of inflammation and immune responses influenced by cortical activity. It may be beneficial to explore specific biochemical cascades or pathways, employing more refined and sophisticated outcome measurement techniques, and potentially even examining changes at the level of messenger RNA (mRNA) expression. The sample size estimates from this study can serve as a guide for powering these future trials. Moreover, future research should incorporate longer intervention periods and extended follow-up durations, potentially up to six months, with a more pragmatic approach to participant care.

Another strength of the current study is the success in maintaining participant blinding. Over 94% of participants, regardless of their group allocation, believed they had undergone an active chiropractic intervention, demonstrating that blinding was adequately achieved. This is particularly challenging in trials involving manual interventions [64,94], yet it reinforces the conclusion that observed between-group differences were not merely a result of contextual or placebo effects.

## Conclusion

In conclusion, this study demonstrates that 12 weeks of chiropractic care was associated with increased blood BDNF and IL-6 levels and decreased TNF-α levels at 12 weeks, along with reduced blood cortisol and IFN-γ levels at 16 weeks compared with sham care. These findings suggest that chiropractic care may modulate neurotrophic, stress, and inflammatory pathways. While the effects were modest and some confidence intervals were wide, the results provide preliminary evidence for physiological changes beyond musculoskeletal outcomes. Larger and longer-term trials are needed to confirm these findings and to clarify their clinical relevance.

## Supporting information

S1 File Detailed statistical analysis report. This file includes full R code and model outputs.
(ZIP)

## Abbreviations

BDNF: Brain-Derived Neurotrophic Factor
CNS: Central Nervous System
CSMC: Central Segmental Motor Control
CC: Chiropractic Care
CRP: C-Reactive Protein
CD8: Cytotoxic T cells
ELISA: Enzyme-Linked Immunosorbent Assay
IGF-1: Insulin-Like Growth Factor
IL: Interleukin
CD19: Lymphocytes
MGF: Mechano-Growth Factor
CD56: Natural Killer Cells
NGF: Nerve Growth Factor
PFC: Prefrontal Cortex
RCT: Randomized Controlled Trial
SMT: Spinal Manipulative Therapy
CD4: T-helper cells
TNF-α: Tumor Necrosis Factor-Alpha
VAS: Visual Analogue Scale.
