# Supplementary material for: The effects of 12 weeks of chiropractic spinal adjustments on physiological biomarkers in adults: A pragmatic randomized controlled trial
Source: PLoS One. 2025 Dec 11;20(12):e0338730. doi: 10.1371/journal.pone.0338730 (PMC12697962; doi:10.1371/journal.pone.0338730)
Supplement: S1 File — This file includes full R code and model outputs. (ZIP) [file pone.0338730.s001.zip › S1.pdf]

The effects of 12 weeks of chiropractic spinal adjustments on Physiological biomarkers in adults: Supplementary file

Usman Rashid

25-Oct-2025

Contents

1 Hair Cortisol **3**
1.1 Model . . . 3
1.2 Between Group Statistics . . . 4
1.3 Within Group Statistics . . . 4

2 Cortisol **5**
2.1 Model . . . 5
2.2 Between Group Statistics . . . 6
2.3 Within Group Statistics . . . 7

3 BDNF **8**
3.1 Model . . . 8
3.2 Between Group Statistics . . . 9
3.3 Within Group Statistics . . . 9

4 CD8 **11**
4.1 Model . . . 11
4.2 Between Group Statistics . . . 12
4.3 Within Group Statistics . . . 12

5 INFG **14**
5.1 Model . . . 14
5.2 Between Group Statistics . . . 15
5.3 Within Group Statistics . . . 15

6 CD4, CRP, TNF **17**
6.1 Model . . . 18
6.2 Between Group Statistics . . . 19
6.3 Within Group Statistics . . . 19

7 CD19, IL6 **20**
7.1 Model . . . 20
7.2 Between Group Statistics . . . 21
7.3 Within Group Statistics . . . 22

8 CD56 **23**
8.1 Model . . . 23

8.2 Between Group Statistics . . . 24
8.3 Within Group Statistics . . . 24

9 Change over Time 26

10 Sensitivity Ananlysis 26
10.1 Cortisol . . . 27
10.1.1 Between Group Statistics . . . 27
10.1.2 Within Group Change Statistics . . . 27
10.2 BDNF . . . 28
10.2.1 Between Group Statistics . . . 28
10.2.2 Within Group Change Statistics . . . 28
10.3 INFG . . . 29
10.3.1 Between Group Statistics . . . 29
10.3.2 Within Group Change Statistics . . . 29
10.4 CD4, CRP, TNF . . . 30
10.4.1 Between Group Statistics . . . 30
10.4.2 Within Group Change Statistics . . . 30
10.5 CD19, IL6 . . . 31
10.5.1 Between Group Statistics . . . 31
10.5.2 Within Group Change Statistics . . . 31

1 Hair Cortisol

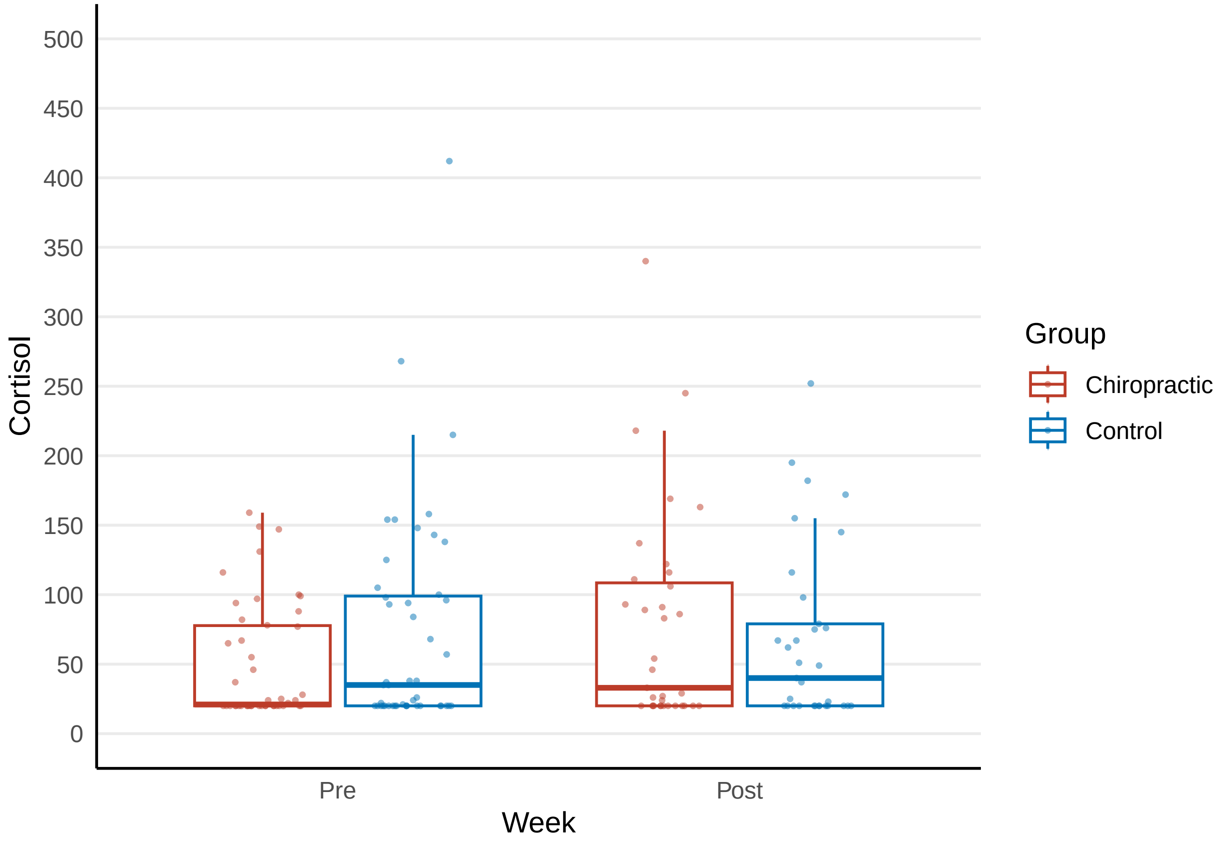

Group	Time	n
Chiropractic	Pre	46
Chiropractic	Post	35
Control	Pre	47
Control	Post	33

1.1 Model

```
mdl.cortisol.hair <- lm ( (Cortisol - CortisolPre) ~ CortisolPre + Group,
                          data.source.hair )
```

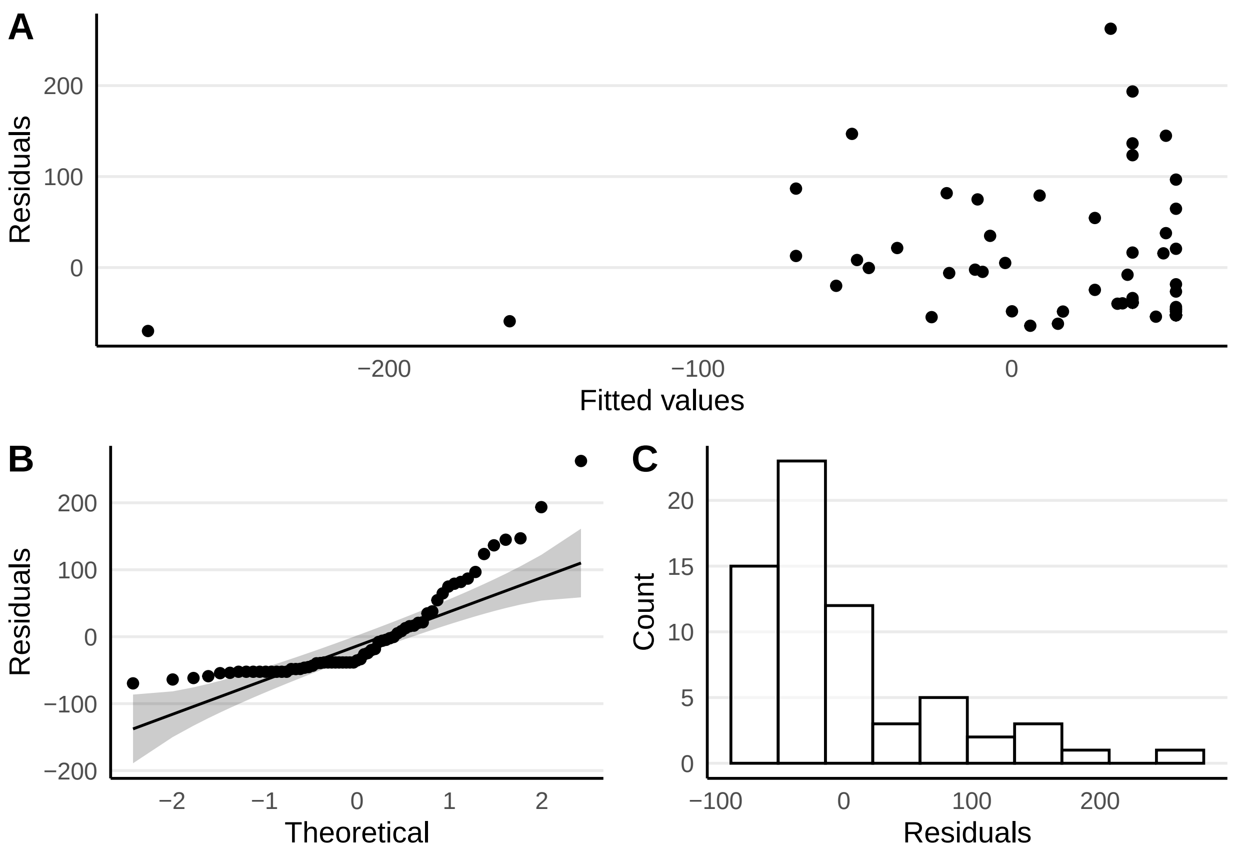


```
Anova Table (Type II tests)

Response: (Cortisol - CortisolPre)
            Sum Sq Df F value    Pr(>F)
CortisolPre 180957  1 36.1323 1.066e-07 ***
Group         3037  1  0.6065    0.4391
Residuals   310508 62
---
Signif. codes:  0 '***' 0.001 '**' 0.01 '*' 0.05 '.' 0.1 ' ' 1
```

1.2 Between Group Statistics

Contrast	Difference±SE [95% CI], SMD	t[df], p-value
Chiropractic - Control	10±20 [-20, 50], 0.1	t[62]=0.779, 0.439

1.3 Within Group Statistics

Group	Estimate±SE [95% CI], SMD	t[df], p-value
Chiropractic	20±10 [-10, 50], 0.23	t[62]=1.841, 0.136
Control	10±10 [-20, 40], 0.09	t[62]=0.674, 0.753

2 Cortisol

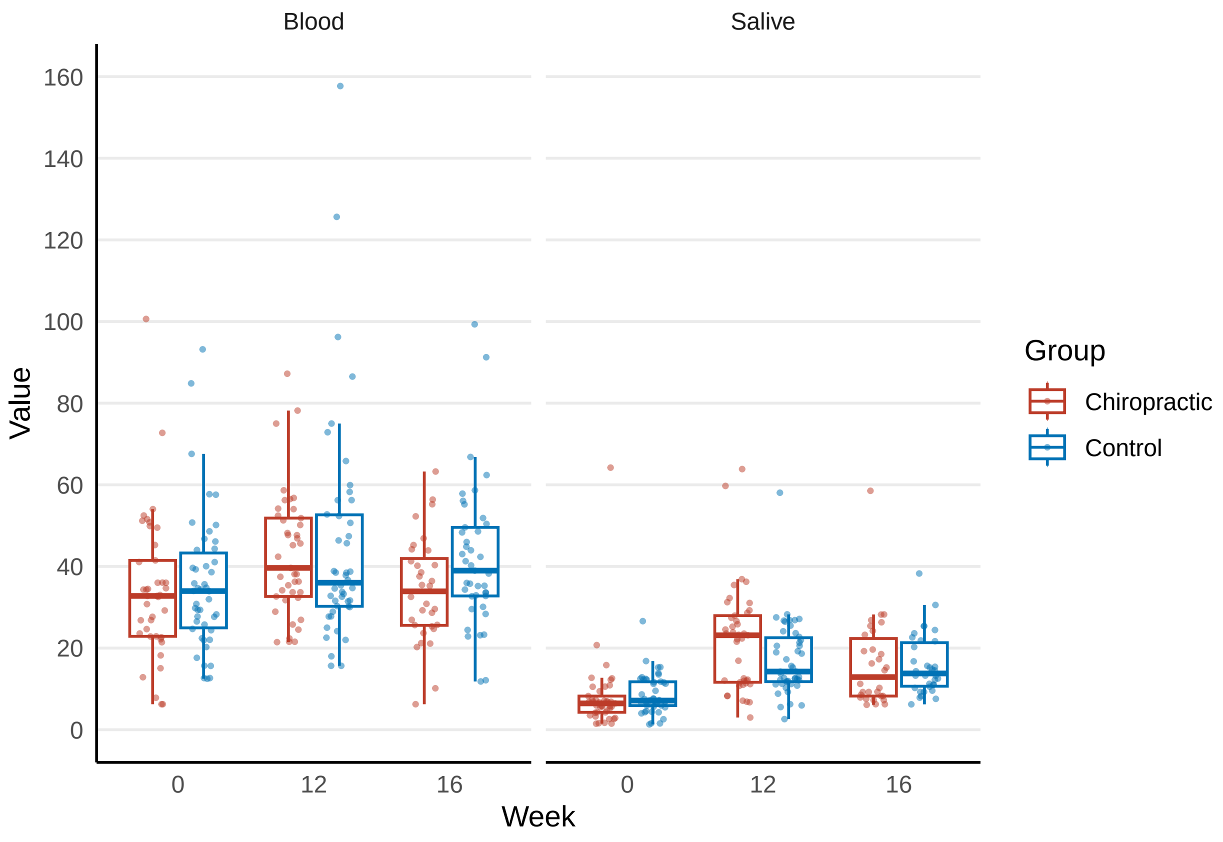

Group	Time	Sample.type	Biomarker	n
Chiropractic	0	Blood	Cortisol	41
Chiropractic	0	Salive	Cortisol	41
Chiropractic	12	Blood	Cortisol	41
Chiropractic	12	Salive	Cortisol	41
Chiropractic	16	Blood	Cortisol	32
Chiropractic	16	Salive	Cortisol	30
Control	0	Blood	Cortisol	46
Control	0	Salive	Cortisol	46
Control	12	Blood	Cortisol	46
Control	12	Salive	Cortisol	46
Control	16	Blood	Cortisol	41
Control	16	Salive	Cortisol	34

2.1 Model

```
mdl.cortisol <- lmer ( (Value - Baseline) ~ Baseline + Group*Time*Sample.type +
                       (Sample.type|ID),
                      data.source.wide.wb |> subset(Biomarker == 'Cortisol') )
```

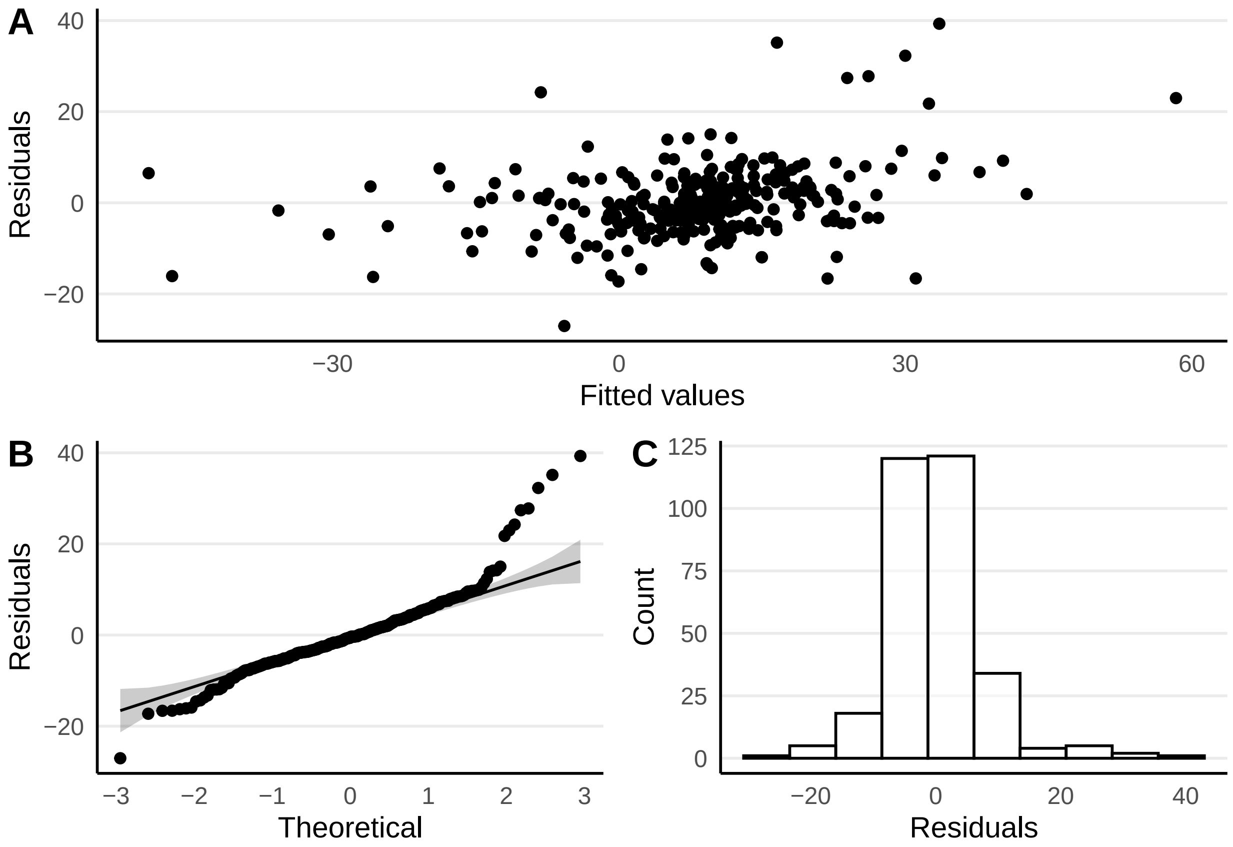


```
Analysis of Deviance Table (Type II Wald chisquare tests)

Response: (Value - Baseline)
                         Chisq Df Pr(>Chisq)
Baseline               48.6133  1  3.118e-12 ***
Group                   0.4452  1    0.50464
Time                   15.5397  1  8.079e-05 ***
Sample.type            17.5562  1  2.789e-05 ***
Group:Time              6.3713  1    0.01160 *
Group:Sample.type       3.8872  1    0.04866 *
Time:Sample.type        1.4140  1    0.23439
Group:Time:Sample.type  0.4709  1    0.49258
---
Signif. codes:  0 '***' 0.001 '**' 0.01 '*' 0.05 '.' 0.1 ' ' 1
```

2.2 Between Group Statistics

Contrast	Time	Sample.type	Difference±SE [95% CI], SMD	t[df], p-value
Chiropractic - Control	12	Blood	-2±4 [-9, 6], -0.05	t[109.5]=-0.488, 0.627
Chiropractic - Control	16	Blood	-9±4 [-17, -1], -0.2	t[125.9]=-2.286, 0.024
Chiropractic - Control	12	Salive	5±2 [0, 10], 0.16	t[154.1]=2.024, 0.045
Chiropractic - Control	16	Salive	1±3 [-5, 6], 0.02	t[176.8]=0.286, 0.775

2.3 Within Group Statistics

Group	Time	Sample.type	Estimate±SE [95% CI], SMD	t[df], p-value
Chiropractic	12	Blood	16±3 [9, 23], 0.51	t[115.3]=5.453, <0.001
Control	12	Blood	18±3 [11, 24], 0.58	t[117.2]=6.271, <0.001
Chiropractic	16	Blood	6±3 [-1, 13], 0.18	t[135.4]=2.039, 0.085
Control	16	Blood	15±3 [9, 22], 0.47	t[124.9]=5.28, <0.001
Chiropractic	12	Salive	6±2 [1, 10], 0.18	t[213.9]=2.698, 0.015
Control	12	Salive	1±2 [-4, 5], 0.03	t[215.6]=0.404, 0.902
Chiropractic	16	Salive	0±2 [-5, 6], 0.01	t[249.2]=0.172, 0.981
Control	16	Salive	0±2 [-5, 5], -0.01	t[237.5]=-0.172, 0.981

3 BDNF

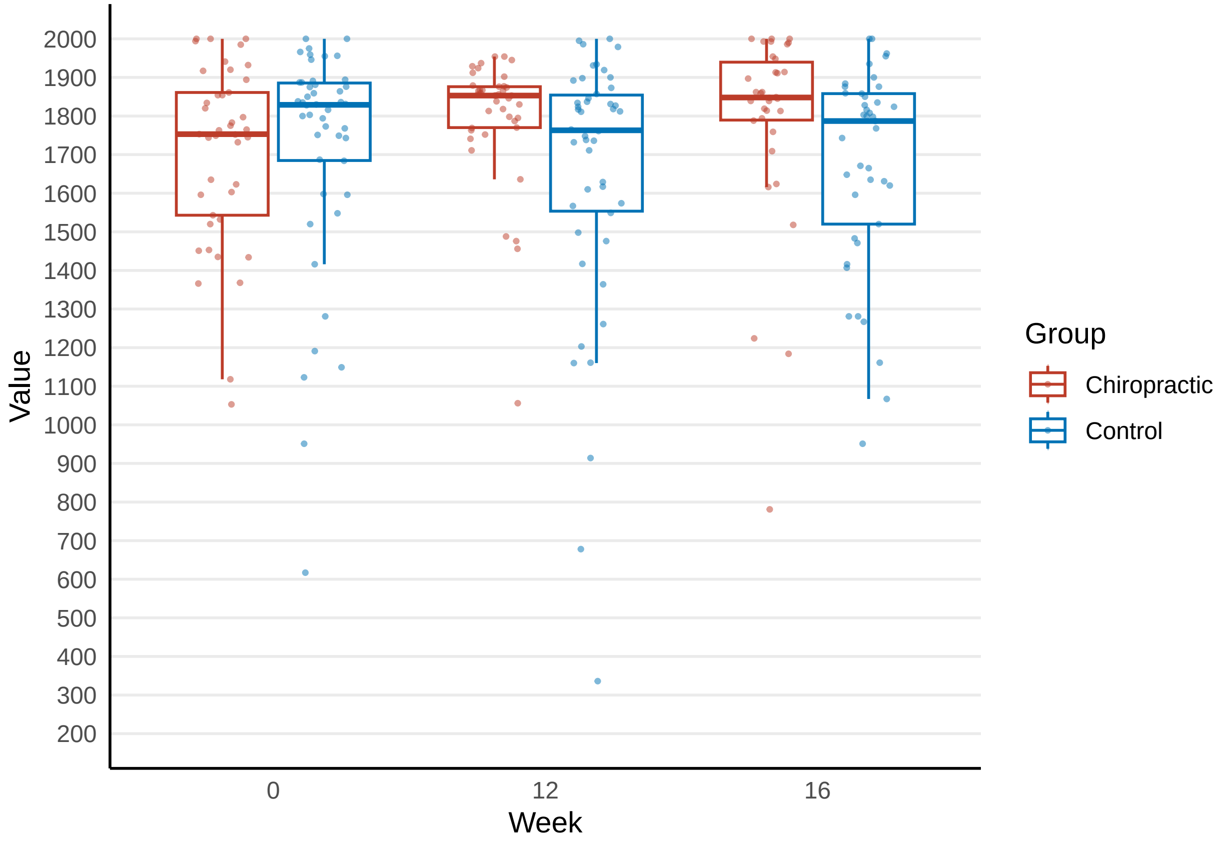

Group	Time	Sample.type	Biomarker	n
Chiropractic	0	Blood	BDNF	41
Chiropractic	12	Blood	BDNF	41
Chiropractic	16	Blood	BDNF	34
Control	0	Blood	BDNF	46
Control	12	Blood	BDNF	46
Control	16	Blood	BDNF	41

3.1 Model

```
mdl.bdnf <- lmer ( (Value - Baseline) ~ Baseline + Group*Time +
                    (1|ID),
                   data.source.wide.wb |> subset(Biomarker == 'BDNF') )
```

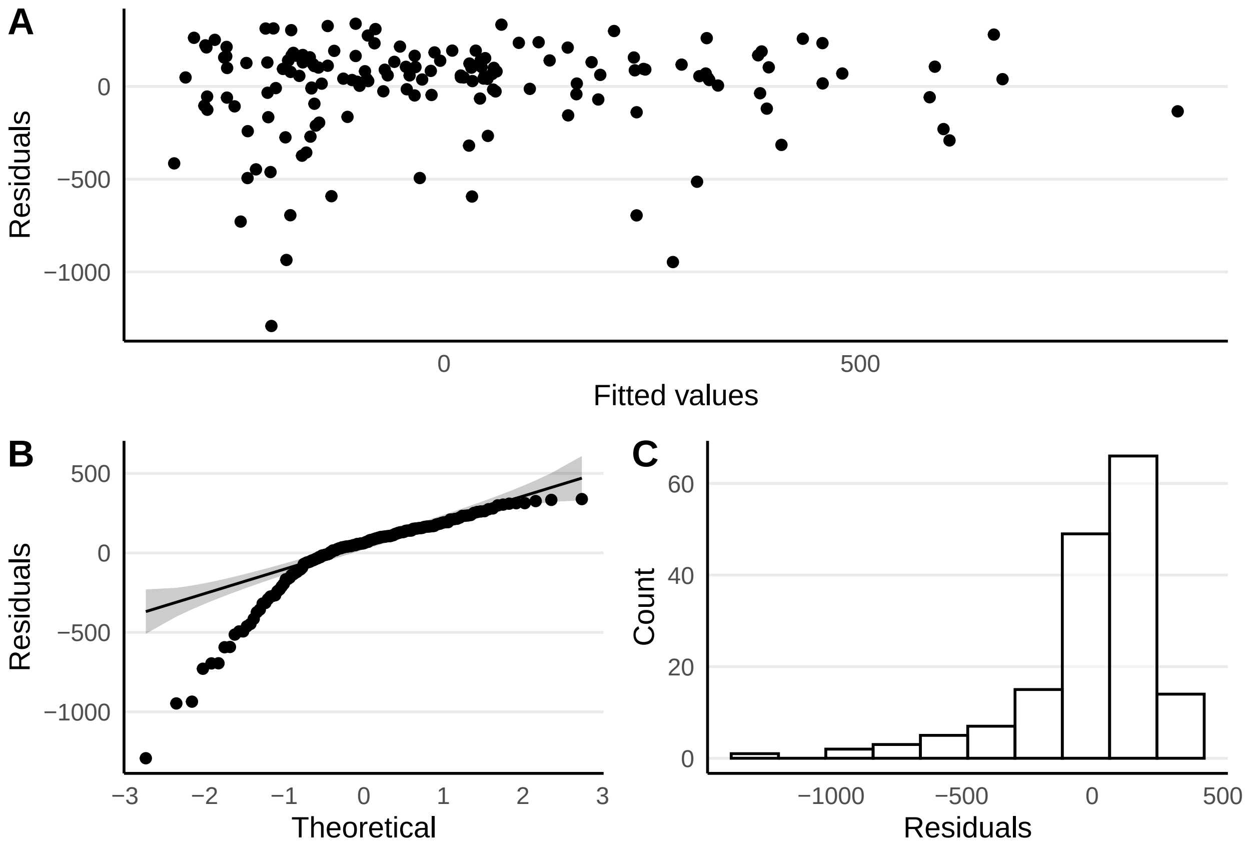


```
Analysis of Deviance Table (Type II Wald chisquare tests)

Response: (Value - Baseline)
             Chisq Df Pr(>Chisq)
Baseline   107.636  1  < 2.2e-16 ***
Group       10.207  1   0.001399 **
Time         0.036  1   0.849607
Group:Time   0.163  1   0.686435
---
Signif. codes:  0 '***' 0.001 '**' 0.01 '*' 0.05 '.' 0.1 ' ' 1
```

3.2 Between Group Statistics

Contrast	Time	Difference±SE [95% CI], SMD	t[df], p-value
Chiropractic - Control	12	150±60 [40, 270], 0.21	t[156.8]=2.65, 0.009
Chiropractic - Control	16	120±60 [0, 250], 0.15	t[156.9]=1.904, 0.059

3.3 Within Group Statistics

Group	Time	Estimate±SE [95% CI], SMD	t[df], p-value
Chiropractic	12	80±40 [-10, 180], 0.16	t[156.8]=1.976, 0.097
Control	12	-70±40 [-160, 20], -0.14	t[156.8]=-1.767, 0.152
Chiropractic	16	70±50 [-30, 180], 0.13	t[156.9]=1.572, 0.222
Control	16	-50±40 [-140, 50], -0.09	t[156.9]=-1.105, 0.469

4 CD8

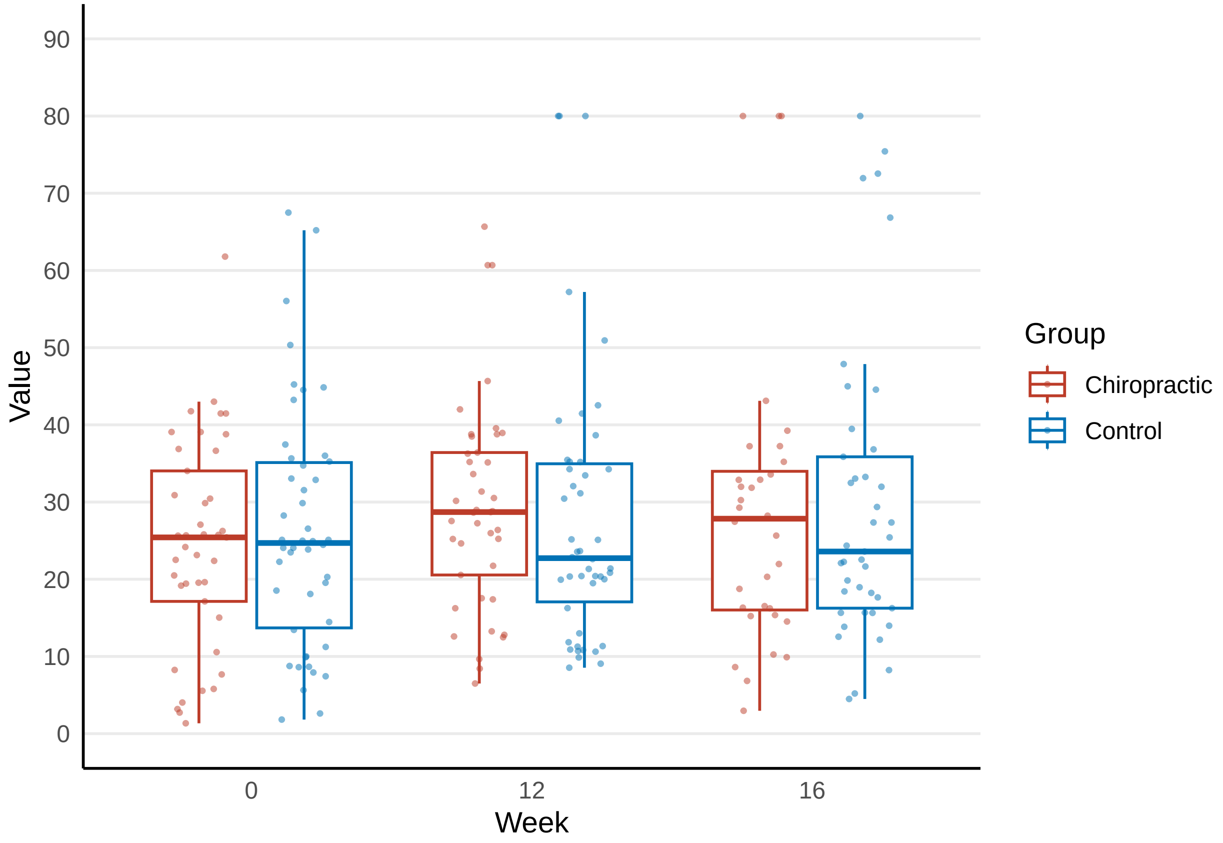

Group	Time	Sample.type	Biomarker	n
Chiropractic	0	Blood	CD8	41
Chiropractic	12	Blood	CD8	41
Chiropractic	16	Blood	CD8	32
Control	0	Blood	CD8	46
Control	12	Blood	CD8	46
Control	16	Blood	CD8	41

4.1 Model

```
mdl.cd8 <- lmer ( (Value - Baseline) ~ Baseline + Group*Time +
                    (1|ID),
                  data.source.wide.wb |> subset(Biomarker %in% c('CD8') ) )
```

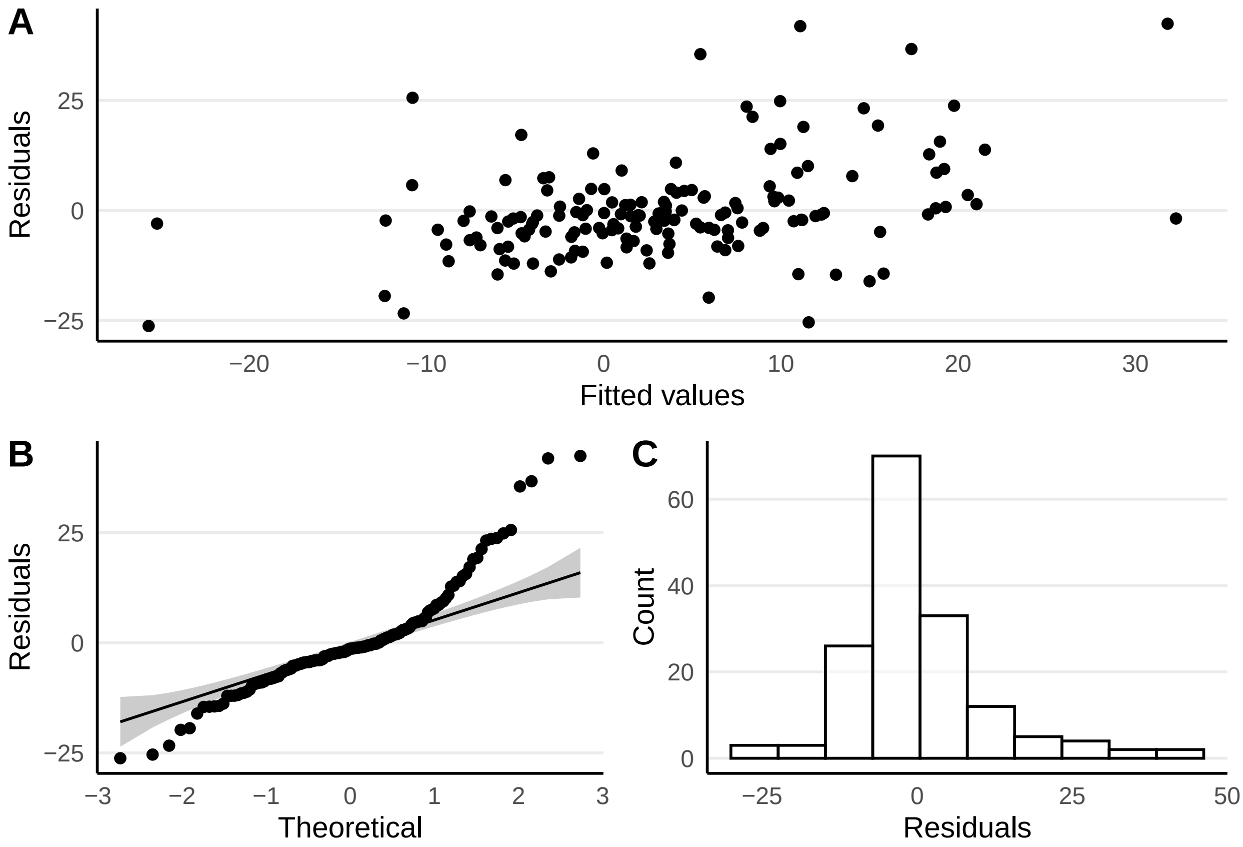


```
Analysis of Deviance Table (Type II Wald chisquare tests)

Response: (Value - Baseline)
             Chisq Df Pr(>Chisq)
Baseline   21.0053  1   4.58e-06 ***
Group       0.2430  1     0.6220
Time        0.0978  1     0.7544
Group:Time  0.2368  1     0.6265
---
Signif. codes:  0 '***' 0.001 '**' 0.01 '*' 0.05 '.' 0.1 ' ' 1
```

4.2 Between Group Statistics

Contrast	Time	Difference±SE [95% CI], SMD	t[df], p-value
Chiropractic - Control	12	2±3 [-4, 9], 0.06	t[141.3]=0.679, 0.498
Chiropractic - Control	16	0±4 [-7, 8], 0.01	t[148.6]=0.069, 0.945

4.3 Within Group Statistics

Group	Time	Estimate±SE [95% CI], SMD	t[df], p-value
Chiropractic	12	4±2 [-1, 10], 0.15	t[141.3]=1.829, 0.134
Control	12	2±2 [-3, 7], 0.08	t[141.4]=0.949, 0.57
Chiropractic	16	4±3 [-2, 10], 0.12	t[150.3]=1.459, 0.272
Control	16	4±2 [-2, 9], 0.13	t[146.2]=1.541, 0.235

5 INFG

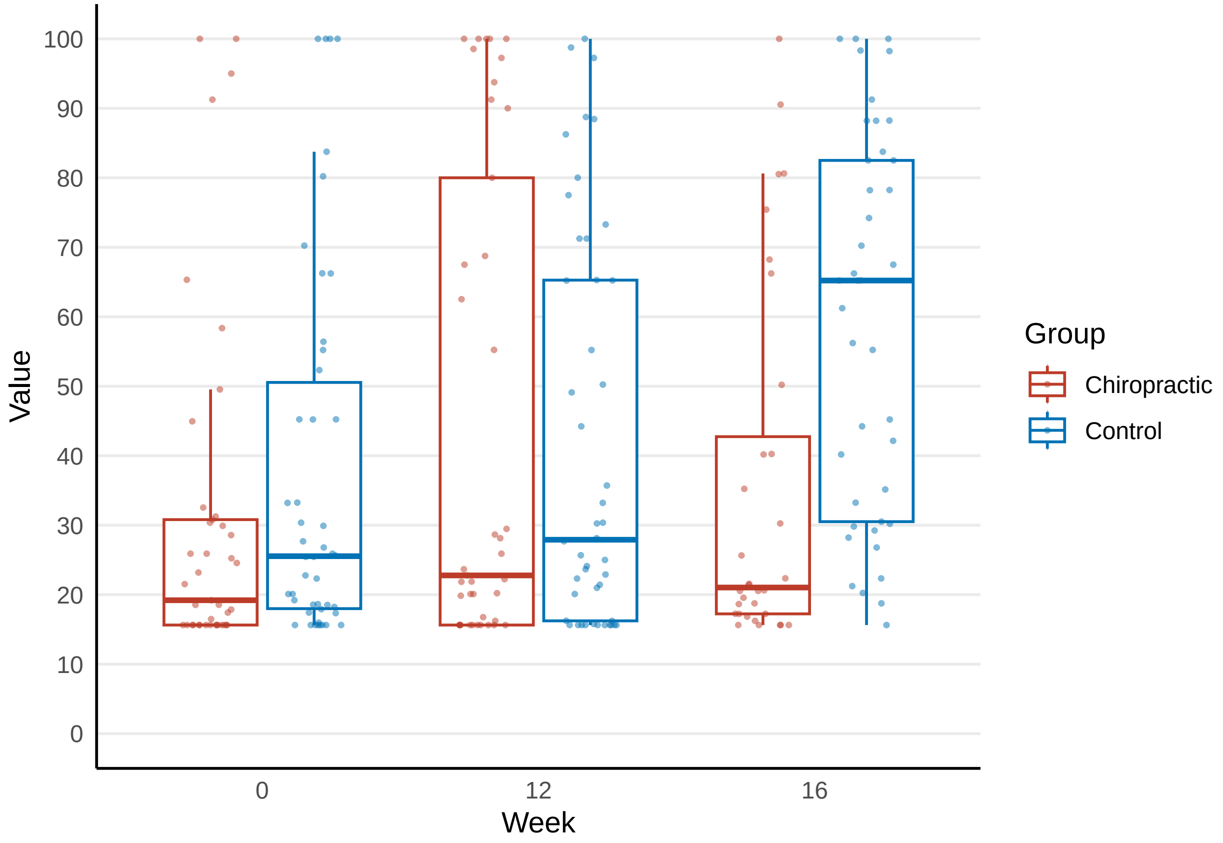

Group	Time	Sample.type	Biomarker	n
Chiropractic	0	Blood	INFG	41
Chiropractic	12	Blood	INFG	41
Chiropractic	16	Blood	INFG	32
Control	0	Blood	INFG	46
Control	12	Blood	INFG	46
Control	16	Blood	INFG	41

5.1 Model

```
mdl.infg <- lmer ( (Value - Baseline) ~ Baseline + Group*Time +
                        (1|ID),
                   data.source.wide.wb |> subset(Biomarker %in% c('INFG') ) )
```

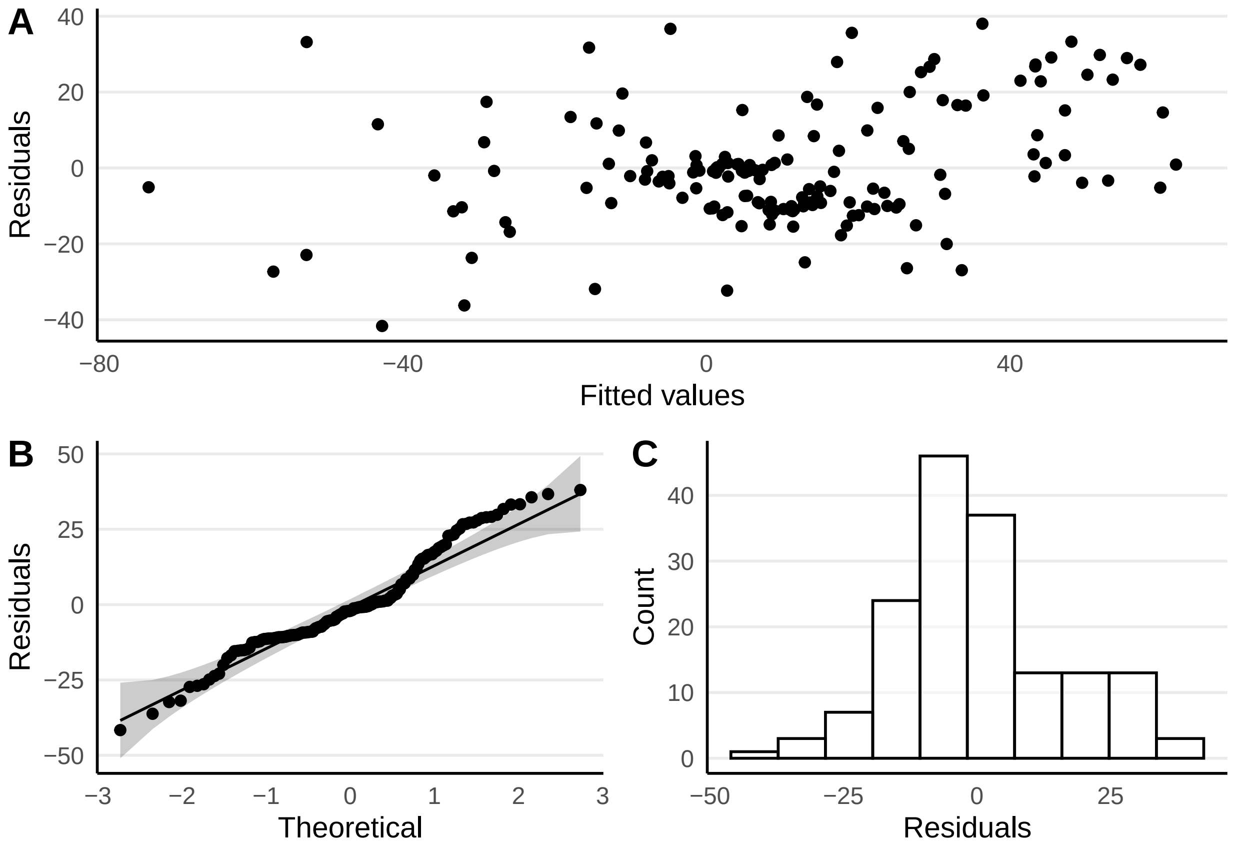


```
Analysis of Deviance Table (Type II Wald chisquare tests)

Response: (Value - Baseline)
             Chisq Df Pr(>Chisq)
Baseline   41.6398  1  1.097e-10 ***
Group       1.5906  1     0.2072
Time        2.1797  1     0.1398
Group:Time 17.0215  1  3.696e-05 ***
---
Signif. codes:  0 '***' 0.001 '**' 0.01 '*' 0.05 '.' 0.1 ' ' 1
```

5.2 Between Group Statistics

Contrast	Time	Difference±SE [95% CI], SMD	t[df], p-value
Chiropractic - Control	12	4±6 [-8, 17], 0.07	t[123.6]=0.732, 0.465
Chiropractic - Control	16	-22±7 [-35, -9], -0.28	t[137]=-3.331, 0.001

5.3 Within Group Statistics

Group	Time	Estimate±SE [95% CI], SMD	t[df], p-value
Chiropractic	12	10±4 [0, 20], 0.21	t[123.7]=2.318, 0.044
Control	12	6±4 [-4, 15], 0.13	t[124]=1.393, 0.305
Chiropractic	16	0±5 [-11, 11], 0.01	t[140.4]=0.076, 0.996
Control	16	22±4 [12, 32], 0.44	t[132.8]=5.089, <0.001

6 CD4, CRP, TNF

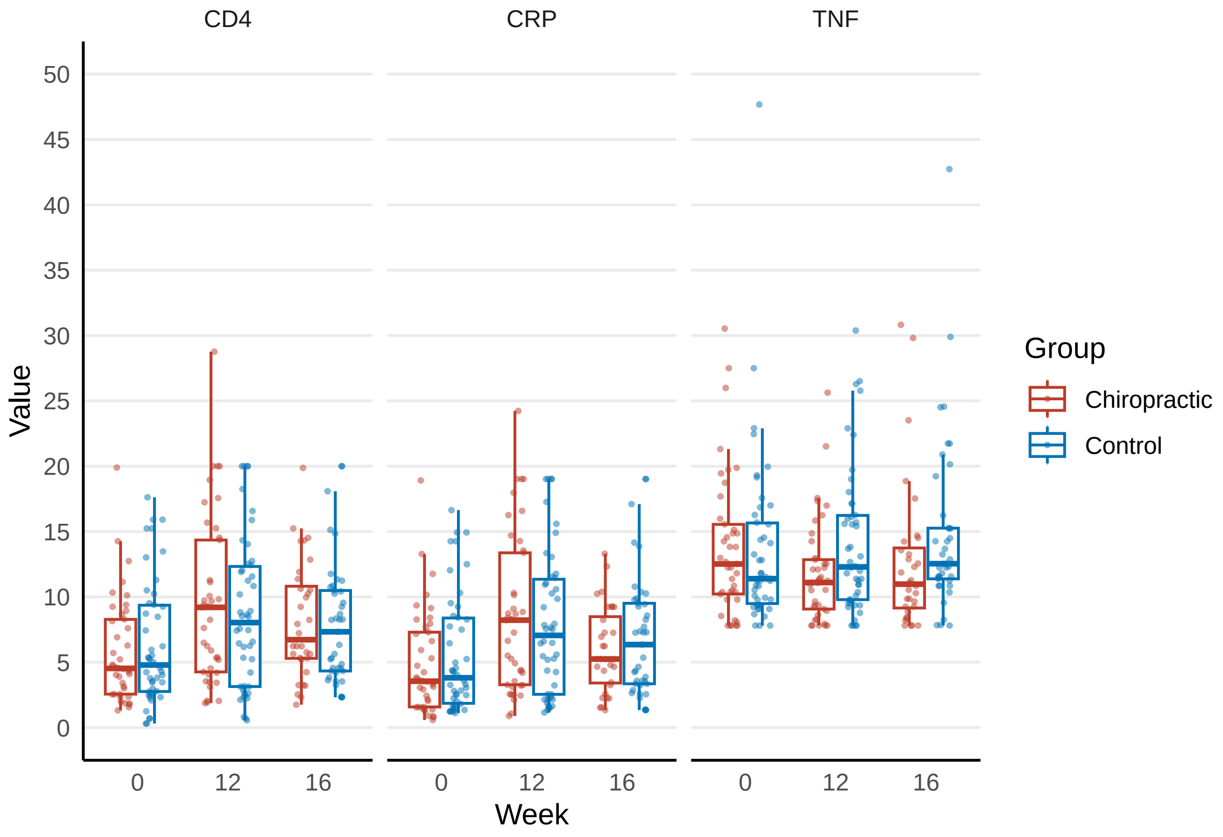

Group	Time	Sample.type	Biomarker	n
Chiropractic	0	Blood	CD4	41
Chiropractic	0	Blood	CRP	41
Chiropractic	0	Blood	TNF	41
Chiropractic	12	Blood	CD4	41
Chiropractic	12	Blood	CRP	41
Chiropractic	12	Blood	TNF	41
Chiropractic	16	Blood	CD4	32
Chiropractic	16	Blood	CRP	32
Chiropractic	16	Blood	TNF	32
Control	0	Blood	CD4	46
Control	0	Blood	CRP	46
Control	0	Blood	TNF	46
Control	12	Blood	CD4	46
Control	12	Blood	CRP	46
Control	12	Blood	TNF	46
Control	16	Blood	CD4	41
Control	16	Blood	CRP	41
Control	16	Blood	TNF	41

6.1 Model

```
mdl.etc <- lmer ( (Value - Baseline) ~ Baseline + Group*Time*Biomarker +
                    (Biomarker|ID),
                  data.source.wide.wb |> subset(Biomarker %in% c('CD4', 'CRP', 'TNF') ) )
```

```
## boundary (singular) fit: see help('isSingular')
```

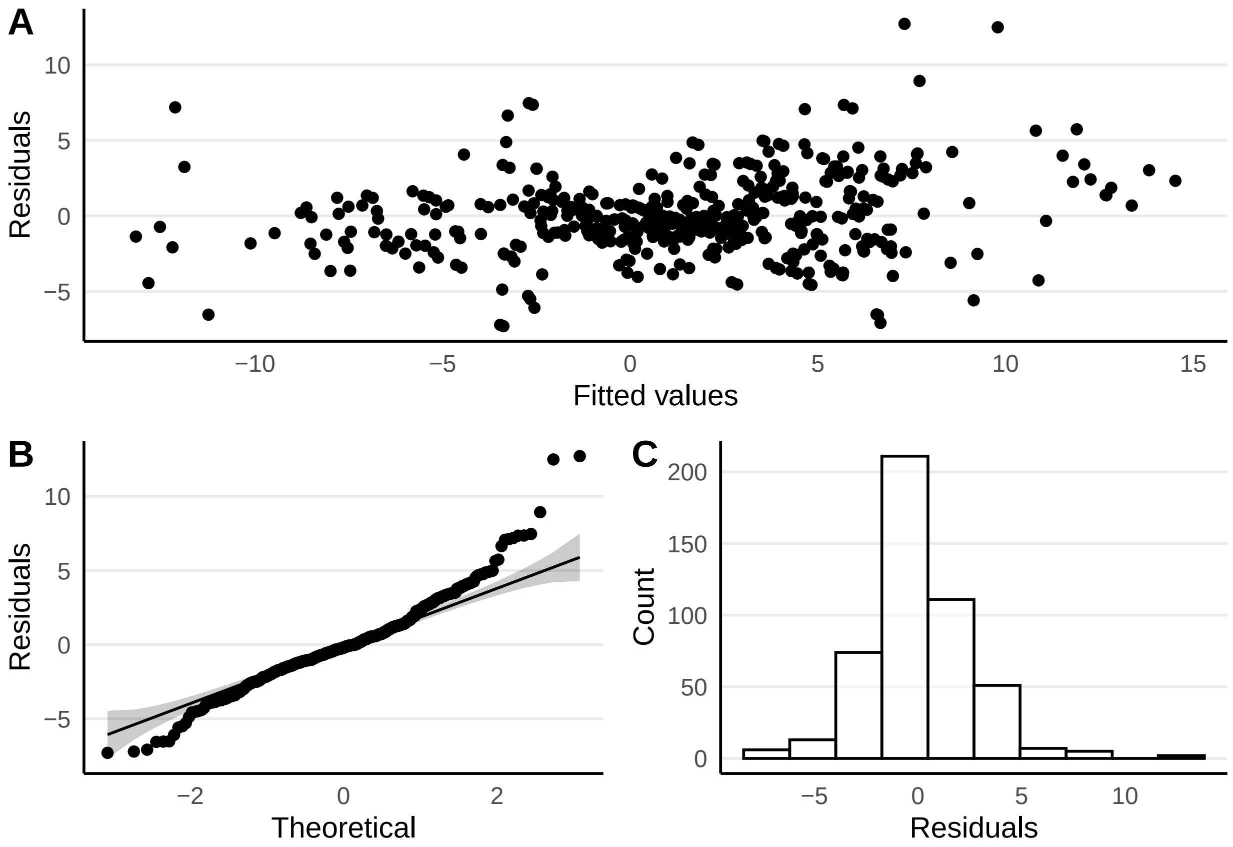


```
Analysis of Deviance Table (Type II Wald chisquare tests)

Response: (Value - Baseline)
                      Chisq Df Pr(>Chisq)
Baseline            88.1976  1  < 2.2e-16 ***
Group                1.6808  1   0.194822
Time                 3.5338  1   0.060130 .
Biomarker            9.9386  2   0.006948 **
Group:Time           1.0829  1   0.298061
Group:Biomarker      6.9251  2   0.031350 *
Time:Biomarker      10.1911  2   0.006124 **
Group:Time:Biomarker 1.0297  2   0.597604
---
Signif. codes:  0 '***' 0.001 '**' 0.01 '*' 0.05 '.' 0.1 ' ' 1
```

6.2 Between Group Statistics

Contrast	Time	Biomarker	Difference±SE [95% CI], SMD	t[df], p-value
Chiropractic - Control	12	CD4	1±1 [-1, 3], 0.09	t[117.6]=0.958, 0.34
Chiropractic - Control	16	CD4	1±1 [-2, 3], 0.05	t[135.6]=0.614, 0.54
Chiropractic - Control	12	CRP	1±1 [-1, 3], 0.08	t[122.1]=0.898, 0.371
Chiropractic - Control	16	CRP	0±1 [-3, 2], -0.04	t[140.9]=-0.421, 0.675
Chiropractic - Control	12	TNF	-2±1 [-4, 0], -0.21	t[124.2]=-2.298, 0.023
Chiropractic - Control	16	TNF	-2±1 [-5, 0], -0.18	t[145.6]=-2.218, 0.028

6.3 Within Group Statistics

Group	Time	Biomarker	Estimate±SE [95% CI], SMD	t[df], p-value
Chiropractic	12	CD4	2.2±0.8 [0.4, 4], 0.24	t[123.8]=2.696, 0.016
Control	12	CD4	1.1±0.8 [-0.6, 2.9], 0.14	t[122.2]=1.496, 0.256
Chiropractic	16	CD4	1.2±0.9 [-0.8, 3.1], 0.11	t[147.2]=1.353, 0.325
Control	16	CD4	0.5±0.8 [-1.3, 2.2], 0.05	t[132.1]=0.592, 0.802
Chiropractic	12	CRP	1.6±0.8 [-0.2, 3.4], 0.18	t[134.3]=2.066, 0.08
Control	12	CRP	0.7±0.7 [-1, 2.4], 0.08	t[131.3]=0.941, 0.575
Chiropractic	16	CRP	-0.6±0.8 [-2.5, 1.3], -0.06	t[157.9]=-0.756, 0.699
Control	16	CRP	-0.2±0.8 [-1.9, 1.5], -0.02	t[141.3]=-0.229, 0.967
Chiropractic	12	TNF	1±0.8 [-0.8, 2.8], 0.11	t[131.9]=1.267, 0.372
Control	12	TNF	3.3±0.8 [1.6, 5], 0.38	t[132.3]=4.376, <0.001
Chiropractic	16	TNF	1.7±0.9 [-0.3, 3.6], 0.15	t[159.3]=1.918, 0.111
Control	16	TNF	4±0.8 [2.3, 5.8], 0.42	t[146.8]=5.116, <0.001

7 CD19, IL6

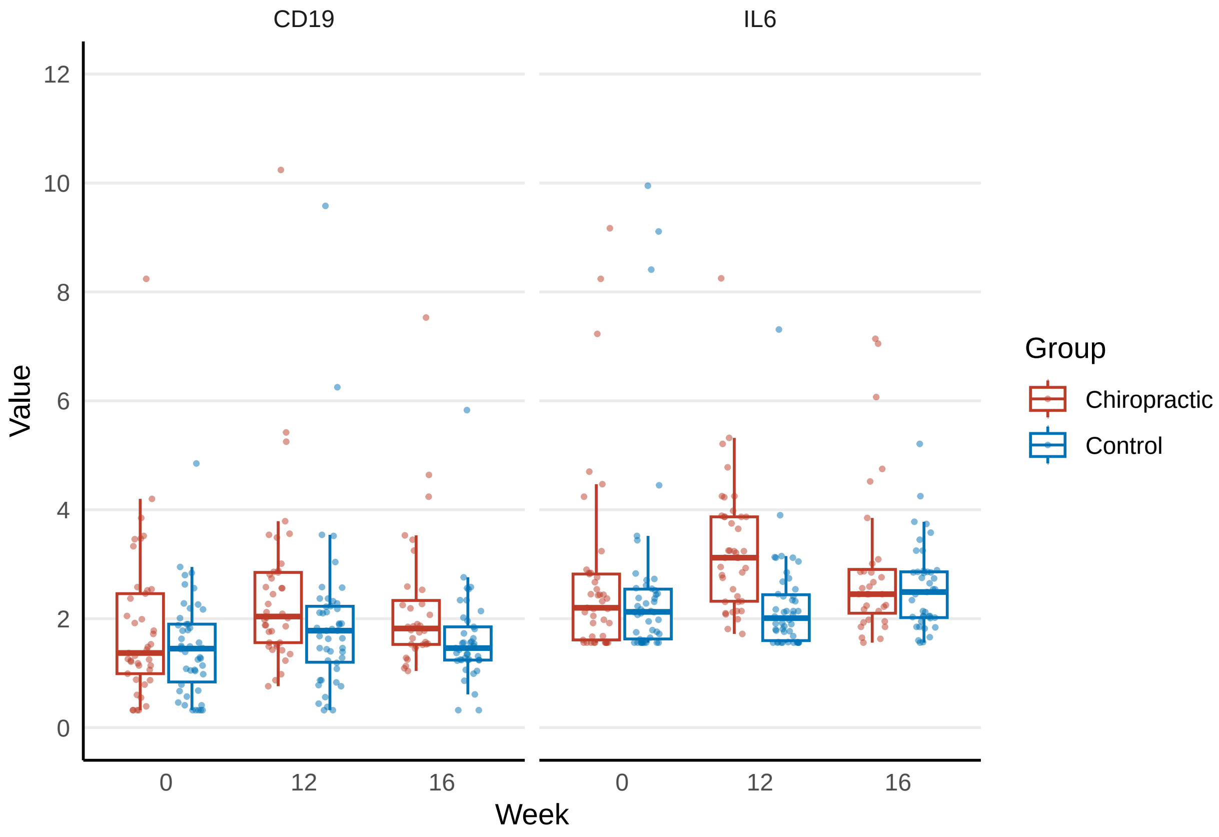

Group	Time	Sample.type	Biomarker	n
Chiropractic	0	Blood	CD19	41
Chiropractic	0	Blood	IL6	41
Chiropractic	12	Blood	CD19	41
Chiropractic	12	Blood	IL6	41
Chiropractic	16	Blood	CD19	32
Chiropractic	16	Blood	IL6	32
Control	0	Blood	CD19	46
Control	0	Blood	IL6	46
Control	12	Blood	CD19	46
Control	12	Blood	IL6	46
Control	16	Blood	CD19	41
Control	16	Blood	IL6	41

7.1 Model

```
mdl.etc2 <- lmer ( (Value - Baseline) ~ Baseline + Group*Time*Biomarker +
                      (Biomarker|ID),
                    data.source.wide.wb |> subset(Biomarker %in% c('CD19', 'IL6') ) )
```

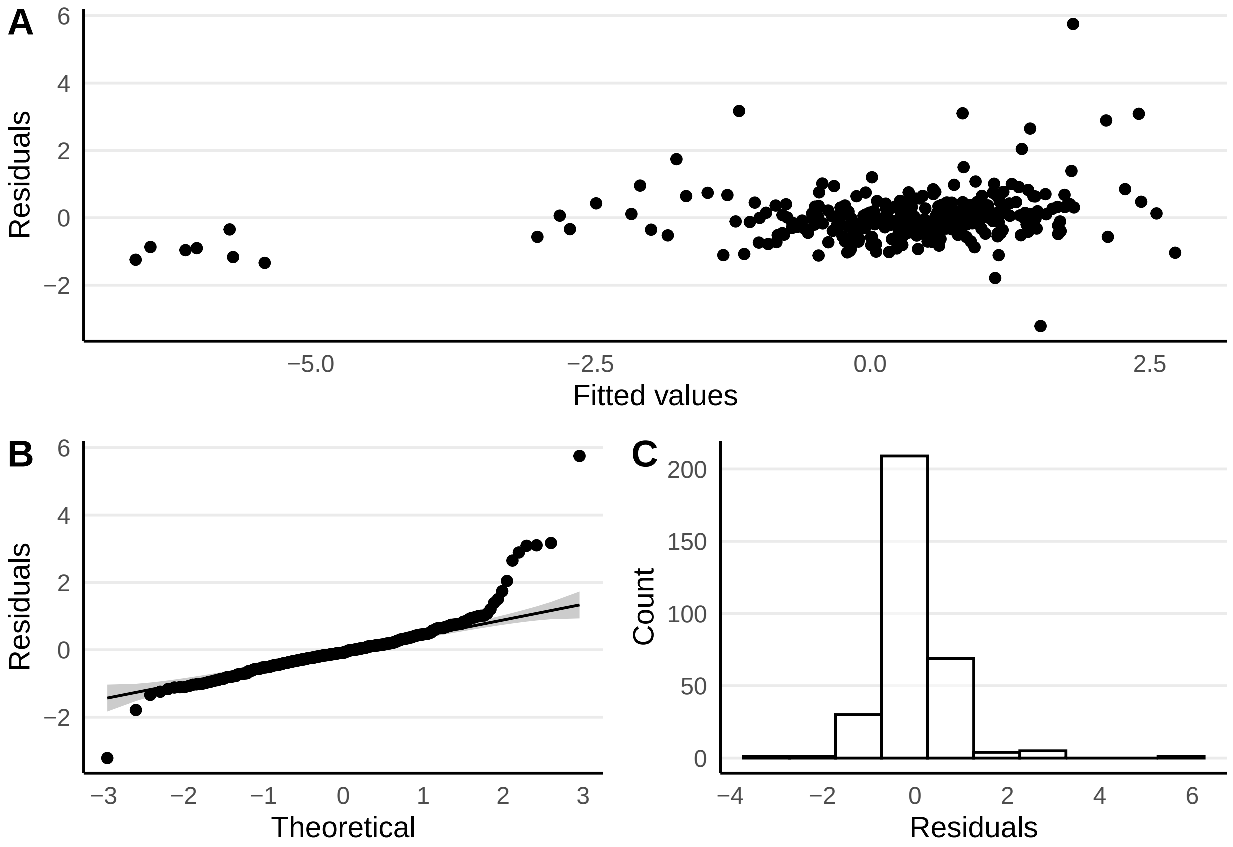


```
Analysis of Deviance Table (Type II Wald chisquare tests)

Response: (Value - Baseline)
                        Chisq Df Pr(>Chisq)
Baseline             226.3017  1  < 2.2e-16 ***
Group                 17.9259  1  2.297e-05 ***
Time                   1.6079  1    0.20478
Biomarker              5.8266  1    0.01579 *
Group:Time             2.3434  1    0.12582
Group:Biomarker        0.6784  1    0.41016
Time:Biomarker         2.3556  1    0.12484
Group:Time:Biomarker   2.4628  1    0.11657
---
Signif. codes:  0 '***' 0.001 '**' 0.01 '*' 0.05 '.' 0.1 ' ' 1
```

7.2 Between Group Statistics

Contrast	Time	Biomarker	Difference±SE [95% CI], SMD	t[df], p-value
Chiropractic - Control	12	CD19	0.5±0.3 [0, 1], 0.15	t[143.6]=1.803, 0.074
Chiropractic - Control	16	CD19	0.5±0.3 [-0.1, 1], 0.13	t[163.3]=1.701, 0.091
Chiropractic - Control	12	IL6	1±0.3 [0.5, 1.5], 0.34	t[144.2]=4.041, <0.001
Chiropractic - Control	16	IL6	0.4±0.3 [-0.2, 0.9], 0.11	t[163]=1.377, 0.17

7.3 Within Group Statistics

Group	Time	Biomarker	Estimate±SE [95% CI], SMD	t[df], p-value
Chiropractic	12	CD19	0.4±0.2 [0, 0.8], 0.19	t[144.1]=2.282, 0.047
Control	12	CD19	0±0.2 [-0.4, 0.4], -0.02	t[148.9]=-0.203, 0.974
Chiropractic	16	CD19	0.1±0.2 [-0.3, 0.6], 0.05	t[168.2]=0.688, 0.742
Control	16	CD19	-0.3±0.2 [-0.7, 0.1], -0.14	t[162.4]=-1.755, 0.156
Chiropractic	12	IL6	1±0.2 [0.6, 1.4], 0.45	t[145.4]=5.391, <0.001
Control	12	IL6	0±0.2 [-0.4, 0.4], -0.01	t[145]=-0.101, 0.994
Chiropractic	16	IL6	0.7±0.2 [0.2, 1.1], 0.25	t[169.9]=3.266, 0.003
Control	16	IL6	0.3±0.2 [-0.1, 0.7], 0.13	t[155.6]=1.628, 0.2

8 CD56

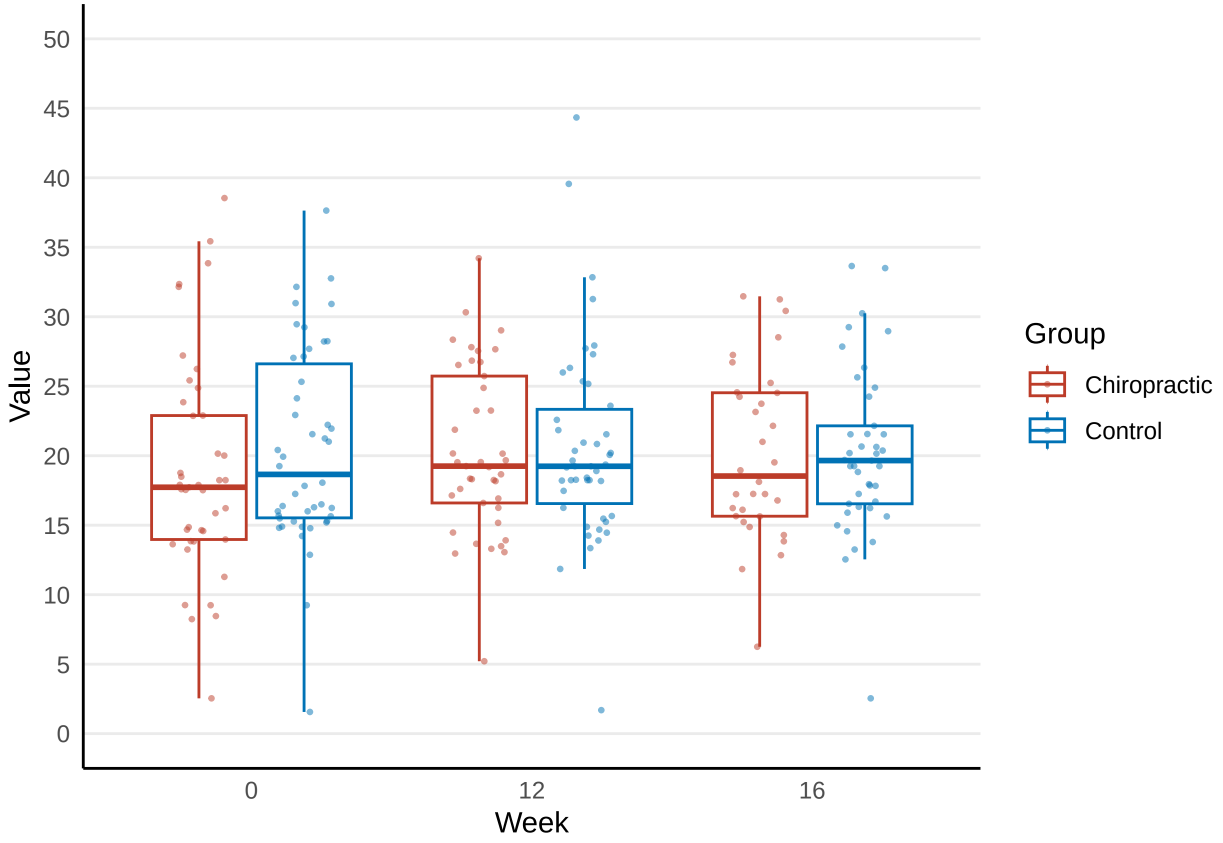

Group	Time	Sample.type	Biomarker	n
Chiropractic	0	Blood	CD56	41
Chiropractic	12	Blood	CD56	41
Chiropractic	16	Blood	CD56	32
Control	0	Blood	CD56	46
Control	12	Blood	CD56	46
Control	16	Blood	CD56	41

8.1 Model

```
mdl.cd56 <- lmer ( (Value - Baseline) ~ Baseline + Group*Time +
                     (1|ID),
                   data.source.wide.wb |> subset(Biomarker %in% c('CD56') ) )
```

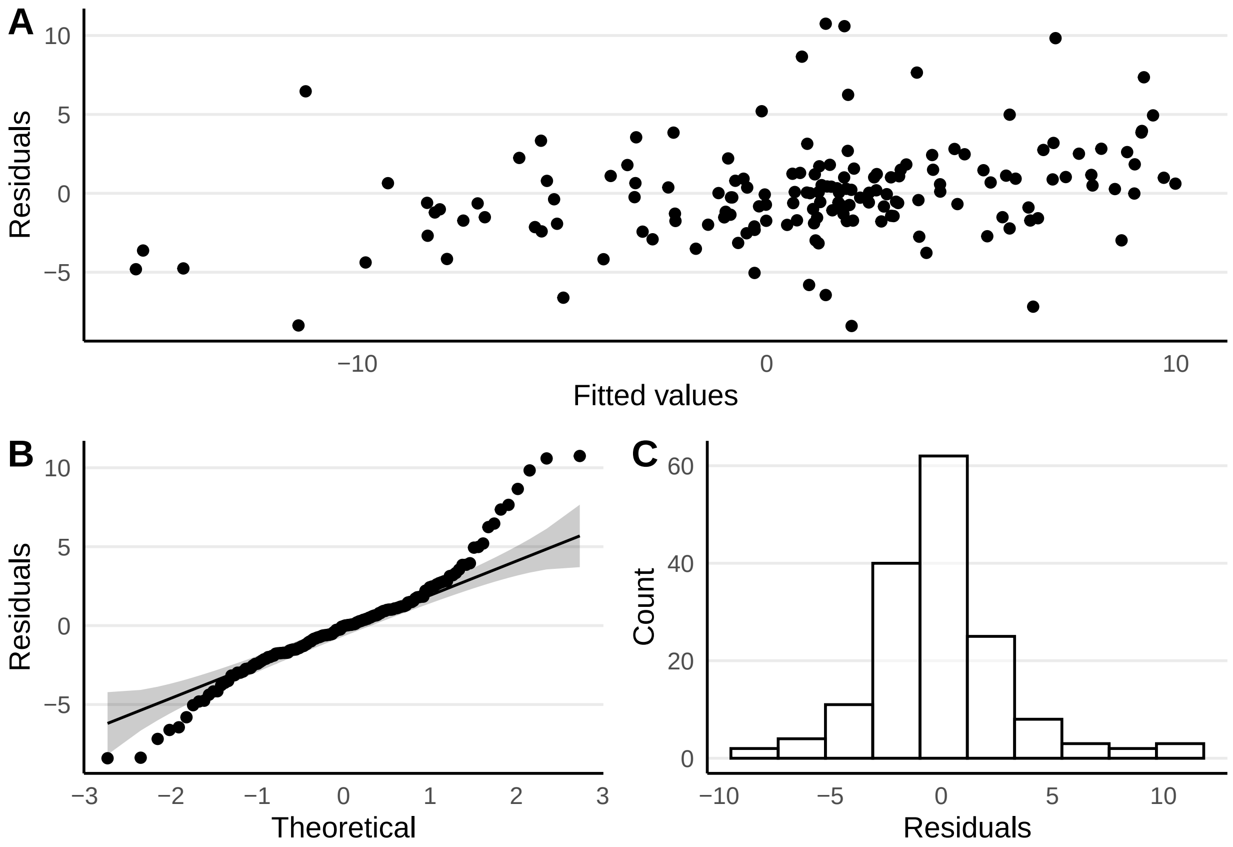


```
Analysis of Deviance Table (Type II Wald chisquare tests)

Response: (Value - Baseline)
             Chisq Df Pr(>Chisq)
Baseline   57.6002  1  3.212e-14 ***
Group       0.4701  1     0.4929
Time        0.0531  1     0.8178
Group:Time  0.3221  1     0.5704
---
Signif. codes:  0 '***' 0.001 '**' 0.01 '*' 0.05 '.' 0.1 ' ' 1
```

8.2 Between Group Statistics

Contrast	Time	Difference±SE [95% CI], SMD	t[df], p-value
Chiropractic - Control	12	0±1 [-2, 3], 0.03	t[125.4]=0.35, 0.727
Chiropractic - Control	16	1±1 [-1, 4], 0.08	t[139.1]=0.885, 0.378

8.3 Within Group Statistics

Group	Time	Estimate±SE [95% CI], SMD	t[df], p-value
Chiropractic	12	1.2±0.9 [-0.8, 3.1], 0.12	t[125.7]=1.35, 0.327
Control	12	0.7±0.8 [-1.1, 2.6], 0.08	t[125.4]=0.914, 0.593
Chiropractic	16	1.7±1 [-0.4, 3.9], 0.15	t[143]=1.793, 0.145
Control	16	0.6±0.9 [-1.4, 2.5], 0.06	t[133.5]=0.671, 0.753

9 Change over Time

```
'geom_line()': Each group consists of only one observation.
i Do you need to adjust the group aesthetic?
```

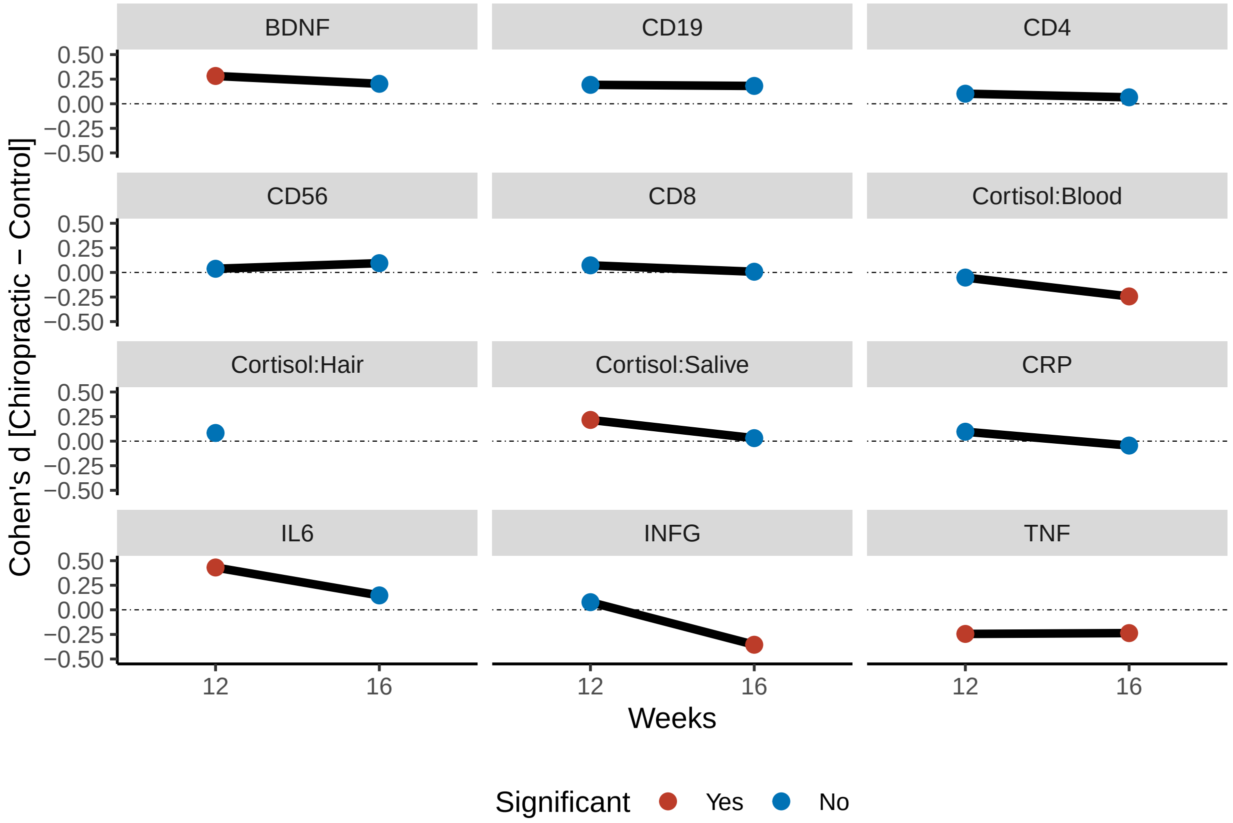

10 Sensitivity Ananlysis

Reconstruction of the primary models on data with missing values imputed with Multivariate Imputations by Chained Equations (MICE).

10.1 Cortisol

10.1.1 Between Group Statistics

Contrast	Time	Sample.type	Difference±SE [95% CI], SMD	t[df], p-value
Chiropractic - Control	12	Blood	-2±4 [-9, 6], -0.04	t[116.6]=-0.484, 0.63
Chiropractic - Control	16	Blood	-8±4 [-16, 0], -0.2	t[96]=-1.973, 0.051
Chiropractic - Control	12	Salive	5±2 [0, 10], 0.14	t[187.4]=1.981, 0.049
Chiropractic - Control	16	Salive	0±3 [-5, 5], 0	t[146.4]=0.017, 0.986

10.1.2 Within Group Change Statistics

Group	Time	Sample.type	Estimate±SE [95% CI], SMD	t[df], p-value
Chiropractic	12	Blood	16±3 [9, 23], 0.5	t[120.5]=5.436, <0.001
Control	12	Blood	18±3 [11, 24], 0.57	t[121.6]=6.24, <0.001
Chiropractic	16	Blood	7±3 [0, 14], 0.22	t[104.8]=2.228, 0.055
Control	16	Blood	15±3 [8, 22], 0.48	t[109.2]=5.014, <0.001
Chiropractic	12	Salive	6±2 [1, 11], 0.19	t[210]=2.734, 0.014
Control	12	Salive	1±2 [-4, 6], 0.03	t[210.2]=0.481, 0.864
Chiropractic	16	Salive	1±2 [-4, 6], 0.04	t[157.7]=0.503, 0.852
Control	16	Salive	1±2 [-4, 6], 0.04	t[133.1]=0.486, 0.862

10.2 BDNF

10.2.1 Between Group Statistics

Contrast	Time	Difference±SE [95% CI], SMD	t[df], p-value
Chiropractic - Control	12	150±60 [30, 280], 0.2	t[155.1]=2.545, 0.012
Chiropractic - Control	16	100±60 [-30, 230], 0.13	t[135.9]=1.548, 0.124

10.2.2 Within Group Change Statistics

Group	Time	Estimate±SE [95% CI], SMD	t[df], p-value
Chiropractic	12	90±40 [-10, 190], 0.16	t[155.1]=1.995, 0.093
Control	12	-70±40 [-160, 30], -0.13	t[155.1]=-1.595, 0.213
Chiropractic	16	50±50 [-50, 160], 0.1	t[133.5]=1.112, 0.464
Control	16	-50±40 [-150, 50], -0.09	t[137.7]=-1.074, 0.488

10.3 INFG

10.3.1 Between Group Statistics

Contrast	Time	Difference±SE [95% CI], SMD	t[df], p-value
Chiropractic - Control	12	4±6 [-8, 17], 0.06	t[128.2]=0.717, 0.475
Chiropractic - Control	16	-18±6 [-31, -5], -0.26	t[117.5]=-2.797, 0.006

10.3.2 Within Group Change Statistics

Group	Time	Estimate±SE [95% CI], SMD	t[df], p-value
Chiropractic	12	11±5 [1, 21], 0.21	t[128.5]=2.41, 0.034
Control	12	6±4 [-3, 16], 0.13	t[128.5]=1.506, 0.251
Chiropractic	16	4±5 [-7, 15], 0.08	t[111.4]=0.82, 0.656
Control	16	22±4 [12, 32], 0.46	t[120.8]=5.024, <0.001

10.4 CD4, CRP, TNF

10.4.1 Between Group Statistics

Contrast	Time	Biomarker	Difference±SE [95% CI], SMD	t[df], p-value
Chiropractic - Control	12	CD4	1±1 [-2, 4], 0.06	t[153.9]=0.763, 0.447
Chiropractic - Control	16	CD4	1±2 [-3, 5], 0.08	t[48.7]=0.558, 0.58
Chiropractic - Control	12	CRP	1±1 [-2, 4], 0.06	t[142.5]=0.69, 0.492
Chiropractic - Control	16	CRP	0±2 [-4, 4], 0.02	t[49.1]=0.146, 0.885
Chiropractic - Control	12	TNF	-2±1 [-5, 0], -0.13	t[200.5]=-1.891, 0.06
Chiropractic - Control	16	TNF	-2±2 [-5, 2], -0.13	t[58.9]=-0.97, 0.336

10.4.2 Within Group Change Statistics

Group	Time	Biomarker	Estimate±SE [95% CI], SMD	t[df], p-value
Chiropractic	12	CD4	2±1 [0, 4], 0.17	t[156]=2.173, 0.062
Control	12	CD4	1.1±0.9 [-1, 3.3], 0.1	t[155.4]=1.22, 0.399
Chiropractic	16	CD4	2±3 [-5, 9], 0.19	t[14.9]=0.749, 0.714
Control	16	CD4	1±2 [-3, 5], 0.1	t[37.4]=0.636, 0.778
Chiropractic	12	CRP	2±1 [-1, 4], 0.13	t[145.5]=1.582, 0.218
Control	12	CRP	0.7±0.9 [-1.4, 2.8], 0.06	t[144.8]=0.717, 0.724
Chiropractic	16	CRP	1±3 [-6, 7], 0.06	t[15.1]=0.249, 0.963
Control	16	CRP	0±2 [-3, 4], 0.04	t[37.7]=0.261, 0.958
Chiropractic	12	TNF	1.3±1 [-0.9, 3.5], 0.1	t[188.1]=1.32, 0.341
Control	12	TNF	3.6±0.9 [1.5, 5.7], 0.28	t[187.1]=3.844, <0.001
Chiropractic	16	TNF	3±3 [-5, 10], 0.22	t[15.5]=0.87, 0.637
Control	16	TNF	4±2 [0, 9], 0.42	t[31.7]=2.379, 0.047

10.5 CD19, IL6

10.5.1 Between Group Statistics

Contrast	Time	Biomarker	Difference±SE [95% CI], SMD	t[df], p-value
Chiropractic - Control	12	CD19	0.5±0.3 [-0.2, 1.1], 0.1	t[205.2]=1.377, 0.17
Chiropractic - Control	16	CD19	0.7±0.5 [-0.2, 1.7], 0.19	t[69.3]=1.57, 0.121
Chiropractic - Control	12	IL6	1±0.3 [0.4, 1.7], 0.22	t[197.8]=3.122, 0.002
Chiropractic - Control	16	IL6	0.5±0.5 [-0.4, 1.4], 0.13	t[65.4]=1.081, 0.284

10.5.2 Within Group Change Statistics

Group	Time	Biomarker	Estimate±SE [95% CI], SMD	t[df], p-value
Chiropractic	12	CD19	0.4±0.2 [-0.1, 1], 0.12	t[205.7]=1.703, 0.172
Control	12	CD19	0±0.2 [-0.6, 0.5], -0.01	t[209.7]=-0.195, 0.976
Chiropractic	16	CD19	0.7±0.6 [-0.9, 2.2], 0.23	t[21]=1.05, 0.518
Control	16	CD19	-0.1±0.4 [-1, 0.8], -0.03	t[46.7]=-0.182, 0.979
Chiropractic	12	IL6	1±0.2 [0.4, 1.5], 0.29	t[197.5]=4.115, <0.001
Control	12	IL6	0±0.2 [-0.5, 0.5], -0.01	t[197.6]=-0.131, 0.989
Chiropractic	16	IL6	1±0.6 [-0.5, 2.5], 0.36	t[21.2]=1.637, 0.219
Control	16	IL6	0.5±0.4 [-0.4, 1.4], 0.2	t[44.4]=1.326, 0.346
